# Macroalga-Derived Alginate Oligosaccharide Alters Intestinal Bacteria of Atlantic Salmon

**DOI:** 10.3389/fmicb.2019.02037

**Published:** 2019-09-13

**Authors:** Shruti Gupta, Jep Lokesh, Yousri Abdelhafiz, Prabhugouda Siriyappagouder, Ronan Pierre, Mette Sørensen, Jorge M. O. Fernandes, Viswanath Kiron

**Affiliations:** ^1^Faculty of Biosciences and Aquaculture, Nord University, Bodø, Norway; ^2^CEVA (Centre d’Etude et de Valorisation des Algues), Pleubian, France

**Keywords:** *Salmo salar*, *Laminaria*, gut bacteria, feed additive, microbiota, amplicon sequencing

## Abstract

Prebiotics are substrates intended to sculpt gut microbial communities as they are selectively utilized by the microorganisms to exert beneficial health effects on hosts. Macroalga-derived oligosaccharides are candidate prebiotics, and herein, we determined the effects of *Laminaria* sp.-derived alginate oligosaccharide (AlgOS) on the distal intestinal microbiota of Atlantic salmon (*Salmo salar*). Using a high-throughput 16S rRNA gene amplicon sequencing technique, we investigated the microbiota harbored in the intestinal content and mucus of the fish offered feeds supplemented with 0.5 and 2.5% AlgOS. We found that the prebiotic shifts the intestinal microbiota profile; alpha diversity was significantly reduced with 2.5% AlgOS while with 0.5% AlgOS the alteration occurred without impacting the bacterial diversity. Beta diversity analysis indicated the significant differences between control and prebiotic-fed groups. The low supplementation level of AlgOS facilitated the dominance of Proteobacteria (including *Photobacterium phosphoreum*, *Aquabacterium parvum*, *Achromobacter insolitus*), and Spirochaetes (*Brevinema andersonii*) in the content or mucus of the fish, and few of these bacteria (*Aliivibrio logei, A. parvum*, *B. andersonii, A. insolitus*) have genes associated with butyrate production. The results indicate that the low inclusion of AlgOS can plausibly induce a prebiotic effect on the distal intestinal microbiota of Atlantic salmon. These findings can generate further interest in the potential of macroalgae-derived oligosaccharides for food and feed applications.

## Introduction

Prebiotics, recently defined by Gibson et al. (2017) are “substrates that are selectively utilized by the host microorganisms conferring a health benefit.” They are intended to evoke beneficial effects on the host through microbial manipulation and the entailing microbial metabolite production. Studies that employed molecular-based methods have provided evidence on the selective effect of prebiotics; they affect certain (e.g., *Bifidobacterium, Anaerostipes*, and *Bilophila*) but not all microorganisms (Vandeputte et al., 2017). Prebiotics such as the non-digestible oligosaccharides are not digested in the gastrointestinal tract (GIT) using host enzymes (Den Besten et al., 2013). The host lacks such enzymes, but certain gut bacteria ferment the carbohydrates into bacterial bioactive metabolites, such as short-chain fatty acids, SCFAs (Flint et al., 2012). One of the main SCFAs, butyrate, benefits the host health by providing energy to colonocytes, and by maintaining mucosal integrity and immune homeostasis (O’Keefe, 2016). Despite the evidence on potential benefits of prebiotics, noted in the aforementioned mammalian studies, their effects on the intestinal microbiota of farmed salmonids have not been thoroughly investigated.

Prebiotics are gradually gaining ground in aquaculture, as feed additives that can alter the gut microbiota and positively affect the host metabolism. The most common prebiotics supplemented in aquafeeds include fructooligosaccharides (FOS), short-chain fructooligosaccharides (scFOS), mannanoligosaccharides (MOS) and few others, as reviewed by Ringø et al. (2010). In fish, prebiotics modulate non-specific immune responses by modifying the gut microbial community, improving mineral uptake and increasing fermentation products (Burr et al., 2005), all of which contribute to improved disease resistance. FOS has been shown to enhance feed efficiency and energy retention in blunt snout bream, *Megalobrama amblycephala* (Wu et al., 2013). scFOS improved specific growth rate and daily feed intake of hybrid tilapia, *Oreochromis aureus × O. niloticus* (Hui-Yuan et al., 2007). Dietary MOS modulated the intestinal microbiota and improved the gut morphology of rainbow trout, *Oncorhynchus mykiss* (Dimitroglou et al., 2009). Furthermore, MOS had a positive effect on feed conversion ratio and elevated the lactic acid producing intestinal bacterial community of common carp (Momeni-Moghaddam et al., 2015). Nevertheless, studies examining the ability of prebiotics to alter the intestinal microbial population in salmonids, including Atlantic salmon, are relatively few. The feeds of Atlantic salmon, a high-value farmed fish, contain 70% plant-based ingredients (Ytrestøyl et al., 2015); some of these are known to affect the micromorphology of the distal intestine (Uran et al., 2009) and composition of the intestinal microbiota (Reveco et al., 2014), leading to intestinal diseases. In this context, prebiotics can modulate fish health by guiding the intestinal microbiota toward a healthy state. Therefore, understanding the changes in the gut microbiota of Atlantic salmon under the influence of a candidate prebiotic is important.

Alginate oligosaccharide (AlgOS), a macroalga product, is suggested as a candidate prebiotic agent because it can promote the host health by favoring the beneficial microorganisms in their gut (Wang et al., 2006). This carbohydrate influences the beneficial gut microflora in *Fenneropenaeus indicus*, Indian major shrimp (Kokilam et al., 2016) and modulates the intestinal microbiota of flat fish (*Solea senegalensis*) (Tapia-Paniagua et al., 2010). Low molecular weight sodium alginate combined with kefir is found to stimulate immunity, disease resistance, and growth performance of Nile tilapia (*Oreochromis niloticus*) (Van Doan et al., 2017). Even though these reports indicate the influence of sodium alginate on the intestinal microbes and fish health, in-depth studies using new techniques can unravel the effect of the compound on the intestinal microbial ecosystem, which is known to have a direct impact on health. Hence, we investigated the effects of AlgOS on the intestinal microbiota of Atlantic salmon.

## Materials and Methods

### Ethics Statement

The study was approved by the Norwegian Animal Research Authority, FDU (Forsøksdyrutvalget ID-8002), and the fish handling and sampling procedures were in accordance with the authorized protocols of FDU.

### Test Product

AlgOS, a candidate prebiotic derived from the macroalga *Laminaria* sp., was obtained from Centre d’Etude et de Valorisation des Algues (CEVA), Pleubian, France. Commercial-grade sodium alginate, Satialgine S 60 NS (Cargill, France) was depolymerized to produce the oligomeric form of sodium alginate. Depolymerization was performed using an enzymatic process based on bacterial alginate-lyase, as described in the patent EP0979301 B1.

### Experimental Fish and Feeding

This study of intestinal microbiota of Atlantic salmon (*Salmo salar*) was part of a 9-week feeding trial conducted at the Research station, Nord University, Bodø. The fish (average weight 185.7 g) were maintained in 800 L tanks of a flow-through seawater system. There were 3 study groups (5 replicate tanks/group); fish of a particular group were offered one of the following feeds: low AlgOS inclusion (0.5 g/100 g – AlgOS-L) or high AlgOS inclusion (2.5 g/100 g – AlgOS-H) or without AlgOS (Control – C). Fish were fed twice daily, between 8:00–9:00 and 14:00–15:00, using automatic feeders (Arvo Teck, Finland). The feed intake was 0.7% BW day^–1^ for all the groups. The water flow rate, temperature and O_2_ levels in the tanks were 1000 L/h, 6.8–7.5°C and above 90%, respectively. A photoperiod of 24:0 L:D was maintained throughout the feeding trial.

### Sampling

At the end of the 9-week feeding period, the fish were sampled after euthanizing them with an overdose (160 mg/L) of MS222 tricaine methanesulfonate (Argent Chemical Laboratories, Redmond, WA, United States). The body surface of the fish was swiped with 70% ethanol before dissection, and the GIT was aseptically removed from the abdominal cavity. The distal intestinal (DI) region was separated from the GIT, the content samples were collected (*n* = 25) using sterile forceps, and then the surface mucus was collected (*n* = 25) using a sterile glass slide by carefully scraping the inner surface. The collected samples were transferred to cryotubes, snap frozen in liquid nitrogen and later stored at −80°C.

Samples were also taken from the fish rearing system to gather information of environmental microbiota. The inlet water (*n* = 1) of the flow-through system as well as the water from each tank (*n* = 5) were collected (1 L) and filtered using a 0.2 μm pore-size filter (Pall Corporation, Hampshire, United Kingdom). Furthermore, biofilm samples from the tank walls (*n* = 5) were scraped and collected in cryotubes. These samples were also flash frozen in liquid nitrogen and stored at –80*°*C.

### DNA Extraction, PCR Amplification, Amplicon Library Construction, and Sequencing

Genomic DNA was extracted from all samples (except water filter samples) using the Quick-DNA^TM^ Fecal/Soil Microbe 96 kit (Zymo Research, Irvine, CA, United States). Genomic DNA from the water filter samples was extracted using Metagenomic DNA Isolation kit for water (Epicentre Biotechnologies, Madison, WI, United States), according to the manufacturer’s instructions. The quality of the extracted DNA was checked on 1.2% (w/v) agarose gel and the DNA concentration was quantified using the Qubit 3.0 fluorometer (Life Technologies, Carlsbad, United States).

The V3–V4 region of the bacterial 16S rRNA gene was targeted for the PCR reactions, based on the dual-index sequencing strategy described by Kozich et al. (2013). PCR reactions were performed in triplicate; each PCR reaction was carried out in 25 μl reaction volume containing 12.5 μl of Kapa HiFi Hot Start PCR Ready Mix (KAPA Biosystems, Woburn, United States), 1.5 μl of each forward and reverse primer (at a final concentration of 100 nM), 3.5 μl of DNAse-free water and 6 μl of DNA template. A negative PCR control without DNA template was also included in the run. The thermocycling conditions included initial denaturation at 95°C for 5 min, followed by 35 cycles of denaturation at 98°C for 30 s, annealing at 58°C for 30 s, extension at 72°C for 45 s, and the final extension performed at 72°C for 2 min. After performing the PCR, the resulting amplicon triplicates were pooled and visualized on 1.2% (w/v) agarose gel. No amplification was observed in the negative PCR control. The amplified products were cut from the gel and purified using the ZR-96 Zymoclean^TM^ Gel DNA Recovery Kit (Zymo Research), following the manufacturer’s instructions and eluted in 15 μl elution buffer. The eluted amplicon libraries (sequencing libraries) were quantified using the KAPA Library Quantification Kit (Kapa Biosystems). For amplicon quantification, each library was serially diluted (1:10,000 and 1:20,000), and qPCR was performed on both of the dilutions. The qPCR reaction mixture consisted of KAPA SYBR FAST qPCR master mix containing the primer premix (12 μl), the diluted library or DNA standard (4 μl) and PCR-grade water (4 μl) for negative control. The Cq values corresponding to the different libraries and the values corresponding to the DNA standards were used to calculate the size-corrected dilution factor for each sample. Each amplicon library was subsequently diluted with low TE buffer (Qiagen, Oslo, Norway) to obtain an equimolar concentration (3 nM) before sequencing. The concentration of the normalized amplicon libraries was validated on the TapeStation (Agilent Biosystems, Santa Clara, United States). The normalized library pool was further diluted to 12 pM, spiked with equimolar 10% Phix control and then paired-end sequencing was performed on an Illumina Miseq sequencing machine (Illumina, San Diego, CA, United States) in 2 runs with inter-run calibrators (i.e., few samples of known sequencing depth) to minimize eventual differences between sequencing runs. FASTQ files from each sample generated from the sequencing machine were used for data analysis.

### Sequence Data Analysis

#### Sequence Data

The quality of the raw reads obtained after high-throughput sequencing was checked using FastQC (Andrews, 2010). Only the forward reads containing the V3 region of the 16S rRNA gene were used for the downstream analysis, since their quality was better than the reverse reads.

#### Construction of Operational Taxonomic Unit (OTU) and Taxonomy Tables, Using the UPARSE Pipeline

The forward reads were processed and analyzed by UPARSE (USEARCH version 9.2.64) software (Edgar, 2013). The reads were truncated to 240 bp, to remove the low-quality base pairs at the 3′-end and then quality-filtered. Furthermore, chimeric sequences were removed using the UCHIME algorithm (Edgar et al., 2011) and then, quality filtered sequences were clustered into operational taxonomic units (OTUs) at 97% sequence similarity threshold. This threshold was chosen because higher cut-off scores may lead to overmerging of up to 15–32% (Mysara et al., 2017). It has also been suggested that 100% is the optimal identity threshold for identifying species using V4 region-targeted sequences (Edgar, 2018c). Taxonomy annotation of short 16S rRNA tags using large databases like SILVA, Greengenes, or the full RDP database may give unreliable predictions (Edgar, 2018a,b). Hence in the present study, we employed the 16S rRNA RDP training set with species names v16. The OTU sequences were assigned to different taxa using the SINTAX algorithm (Edgar, 2016) with a bootstrap cutoff value of 0.5. Afterward, OTUs with a confidence score < 1 at the domain level and the OTUs belonging to the phyla Cyanobacteria and Chlorophyta were removed. The raw 16S rRNA gene sequence data from this study has been deposited in the European Nucleotide Archive (ENA) under the accession number PRJEB27188.

#### Diversity and Composition Analyses

Due to differences in sequencing depth, the OTU table was rarefied to the lowest number (10,604) of sequences per sample to get an even sampling depth to facilitate comparisons between the treatment groups. Furthermore, to employ content and mucus samples from the same fish, only 21 fish from each group were considered for the downstream analyses. Adding on the tank water and biofilm samples, in total 157 samples were used for the downstream analyses.

The R package “iNEXT” v2.0.12 was used to plot the rarefaction and extrapolation curves for the species richness of the intestinal bacterial assemblage (Hsieh et al., 2016). Codes were executed to calculate and generate diversity indices, core and rare microbiota (relative abundance of core taxa and least abundant taxa) and the corresponding plots, using the R packages “phyloseq” v1.22.3 (McMurdie and Holmes, 2013), “microbiome” v1.0.2 (Lahti et al., 2017), and their supporting packages. All the plots were visualized using the functions in “ggplot2” v2.2.1 (Wickham, 2009). The alpha diversity plots were generated for overall species richness (OTU counts), Shannon diversity (effective number of common OTUs), and Simpson diversity (effective number of most abundant OTUs) based on the formula suggested by Jost (2006). For the beta diversity analysis of the content samples, we incorporated weighted UniFrac distance metric because the dispersions of the different groups for this similarity index were similar. In the case of mucus samples, beta diversity was assessed using double principal coordinates analysis (DPCoA) (Fukuyama et al., 2012).

### Statistical Analysis

Statistical analysis was performed using R studio v3.4.3. To detect significant differences in alpha diversity, Kruskal-Wallis test followed by Dunn’s test was employed. As for the beta diversity analysis, the dispersions of the communities were checked using betadisper; thereafter Adonis (PERMANOVA) followed by pairwise comparisons was employed (999 permutations) to understand the significant dissimilarities of the communities. To detect the differentially abundant OTUs in the treatment groups, a tool for microbiome analysis- “ANCOM” v1.1-3 (Mandal et al., 2015) was used, and “Boruta” v5.3.0 R package (Kursa and Rudnicki, 2010) was employed to find the relevant OTUs that caused the differences in the three study groups. Furthermore, Pearson’s Chi-squared test was performed to clarify differences in proportions of the dominant bacterial taxa in the three groups.

### Prediction of Carbohydrate Degradation Capability and Butyrate-Biosynthesis by Significantly Abundant or Relevant Bacteria

Genome mining was performed to detect the occurrence of butyrate-producing genes in the genome of certain bacteria associated with AlgOS-L group. The genomes of these bacteria (selected butyrate producers) in the DI of Atlantic salmon fed AlgOS-L were retrieved from GenBank database (Table 1) and annotated using PROKKA version 1.13 (Seemann, 2014). Butyrate production abilities of the bacteria were assessed by evaluating the distribution of the pathways in each genome, i.e., by understanding the genomic arrangement of butyrate gene clusters suggested by Anand et al. (2016). Genomes were scanned for genes known to be involved in butyrate production and these sequences were then scanned in protein databases using phmmer from HMMER v. 3.1 (Finn et al., 2015) with the default *E*-value parameter cutoff. Phmmer uses a hidden Markov model to predict protein domains by aligning amino acids to databases such as Pfam (Finn et al., 2016). Metabolic pathways associated with SCFA production were constructed using KASS (Moriya et al., 2007). The corresponding pathway IDs were analyzed as described by Vital et al. (2014). dbCAN2 (Zhang et al., 2018) was used to annotate Carbohydrate-Active Enzymes (CAZymes) present in the genomes of the bacteria listed in Tables 1, 2. These CAZymes give an indication of the carbohydrate metabolic capacity of the bacteria (Lombard et al., 2013). Glycoside hydrolases (GH), glycosyl transferase (GT), and polysaccharide lyases (PL) were among the carbohydrate-active enzymes that were scanned in the genomes of the mentioned bacteria.

**TABLE 1:** Details of the sequences used for genome mining and the associated butyrate pathways.

**GenBank ID**	**NZ_AJYJ02000000**	**NZ_FOKY00000000**	**NZ_CP019325**	**NZ_LFRI00000000**	**PVBT00000000**

**Species**	***Aliivibrio logei***	***Brevinema andersonii***	***Achromobacter insolitus***	***Aquabacterium parvum***	***Phyllobacterium myrsinacearum***
**Butyrate production pathways^∗^**					
Pyruvate pathway	** ×**	**×**	**✓**	**✓**	**✓**
4-aminobutyrate pathway	** ×**	**✓**	**×**	** ×**	**✓**
Lysine pathway	**×**	**×**	**×**	**×**	**×**
Glutarate pathway	**✓**	** ×**	**×**	**×**	** ×**

*^∗^× and ✓ indicate absence and presence of a pathway, respectively.*

**TABLE 2:** CAZyme families encoded in the genome of selected bacteria.

**Species^∗^**	***Aliivibrio logei***	***Achromobacter insolitus***	***Aquabacterium parvum***	***Phyllobacterium myrsinacearum***
	GH1	GH3	GH3	GH3
	GH3	GH5	GH13	GH5
	GH13	GH13	GH28	GH13
	PL7	GH31	PL9	
	PL17	GH28		
	PL22	PL1		
# of genes	127	133	114	147

*^∗^The GenBank IDs are given in Table 1.*

## Results

### Sequence Quality, Rarefaction, and Interpretation of Microbiota Analysis

The high-throughput sequencing generated a total of 12,911,308 high-quality raw reads from all the selected samples. The reads were clustered in to 2057 OTUs at 97% identity threshold. These reads were rarified, based on sample-size, to 10,604 reads/sample, and the general adequacy of the sequencing depth was perceived by drawing the rarefaction curves.

To understand the effects of AlgOS on the bacterial diversity and composition of the DI content and mucus, we describe the alterations in the AlgOS-fed fish compared to the control fish. For this, we explain the richness (i.e., counts of individual OTUs, without regard to their abundance) and effective number of OTUs (number equivalents of entropies), and taxonomic compositional differences. Furthermore, relative abundances of the bacterial taxa are reported based on the top 20 abundant (dominant) and low abundant taxa (less abundant compared to the dominant ones). In addition, we present the significant and relevant bacterial communities of the intestinal microbiota. We also predict the butyrate production ability of certain bacteria that were significantly abundant in the AlgOS-L group.

### Diversity and Compositional Differences of the Intestinal and Environmental Microbiota

**FIGURE 1:**
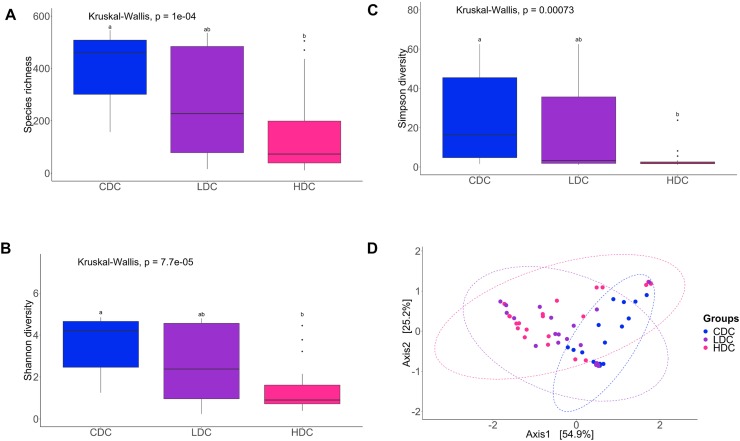
Diversity of the bacterial communities of the distal intestinal content. Boxplots show the species richness **(A)**, Shannon index **(B)**, Simpson index **(C)**. The boxes display the interquartile range (IQR) and shown in the figure are the first (lower hinge) and third (upper hinge) quartiles (25th and 75th percentiles, respectively) and the black horizontal line inside the box represents the median. Whiskers extended from the hinge represent the minimum or maximum values within 1.5 ^∗^ IQR from the hinge, and black dots beyond these values are outliers. Different letters indicate statistically significant differences (*P* < 0.05) between the study groups (CDC, control group; LDC, AlgOS-L group; HDC, AlgOS-H group). Double principal coordinate analysis plot **(D)** shows the beta diversity of the bacterial communities. Ellipses encircle data from a multivariate normal distribution.

**FIGURE 2:**
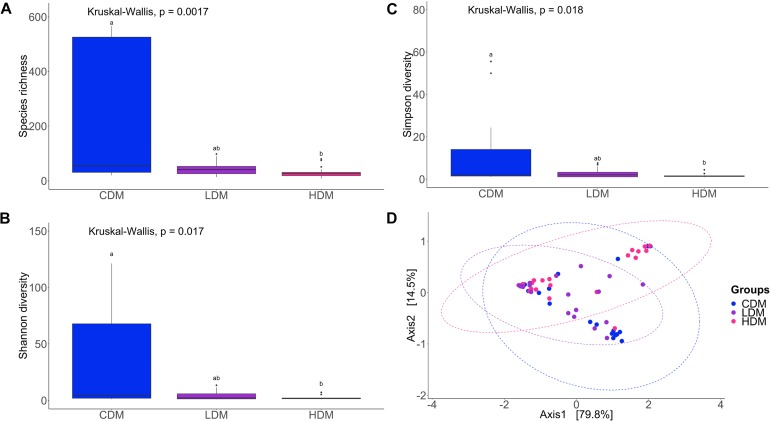
Diversity of the bacterial communities of distal intestinal mucus. Boxplots show the species richness **(A)**, Shannon index **(B)**, Simpson index **(C)**. The boxes display the interquartile range (IQR) and shown in the figure are the first (lower hinge) and third (upper hinge) quartiles (25th and 75th percentiles, respectively) and the black horizontal line inside the box represents the median. Whiskers extended from the hinge represent the minimum or maximum values within 1.5 ^∗^ IQR from the hinge, and black dots beyond these values are outliers Different letters indicate statistically significant differences (*P* < 0.05) between the study groups (CDM, control group; LDM, AlgOS-L group; HDM, AlgOS-H group). Double principal coordinate analysis plot **(D)** shows the beta diversity of the bacterial community. Ellipses encircle data from a multivariate normal distribution.

The species richness of the bacterial community, both in the DI content and mucus, of the AlgOS-H group was significantly lower (*P* < 0.0001 and *P* < 0.001, respectively) compared to the control group (Figures 1A, 2A). The Shannon and the Simpson diversity measures indicated that the effective number of common species and the effective number of dominant species in the AlgOS-H group were significantly lower (Shannon diversity of content *P* < 0.00007 and mucus *P* < 0.017; Simpson diversity of content *P* < 0.0007 and mucus *P* < 0.018) compared to the control group (Figures 1B,C, 2B,C). Faith’s phylogenetic diversity also exhibited a similar trend; the AlgOS-H group had significantly lower (*P* < 0.0001 for content, *P* < 0.002 for mucus) diversity compared to the control group (Supplementary Figures S1A,B). DPCoA, and PCoA based on the weighted UniFrac distance matrix revealed the beta diversity of the DI bacterial communities. We detected significant differences between the control and AlgOS-fed groups (Figure 1D: F-statistic = 5.8676, *R*^2^ = 0.188, *P* < 0.001; and Figure 2D: F-statistic = 3.783, *R*^2^ = 0.113, *P* < 0.005).

The beta diversity analyses were performed for the rearing tank water, and biofilm bacterial communities corresponding to the feeding groups. DPCoA revealed that neither the bacterial communities in the water (Supplementary Figure S2, F-statistic = 0.80906, *R*^2^ = 0.118, *P* > 0.601) nor those in the biofilm (Supplementary Figure S3A, F-statistic = 1.3341, *R*^2^ = 0.1819, *P* > 0.154) were different. DPCoA showed significant differences between biofilm and the fish-associated intestinal bacterial communities (Supplementary Figures S3B–G). Since the DNA extraction from water was performed using a different kit, we have not presented the comparison between the water bacterial communities and the intestinal bacterial communities.

### Abundances of the Intestinal Bacteria

**FIGURE 3:**
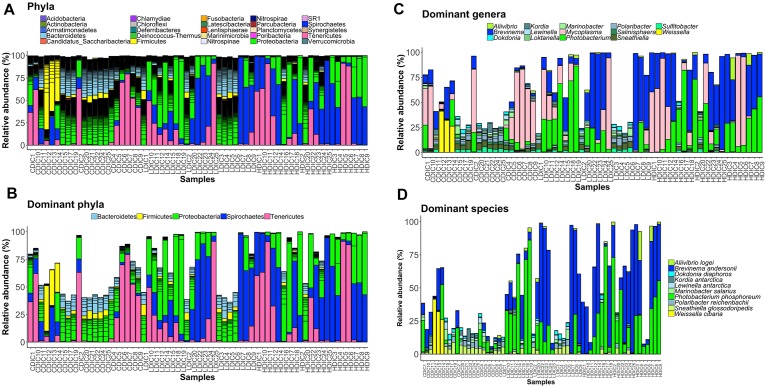
Barplots showing the abundance of the bacterial phyla **(A)**, dominant phyla **(B)**, dominant genera **(C)** and dominant species **(D)** in the distal intestinal content. The height of each bar segment represents the abundance of individual operational taxonomic units (OTUs) stacked in order from greatest to least and separated by a thin black border line. Color codes: Proteobacteria – green, Spirochaetes – dark blue, Bacteroidetes – light blue, Firmicutes – yellow, Tenericutes – magenta. Sample names starting with CDIC are control distal intestinal content, LDIC are AlgOS-L-group distal intestinal content and HDIC are AlgOS-H-group distal intestinal content.

**FIGURE 4:**
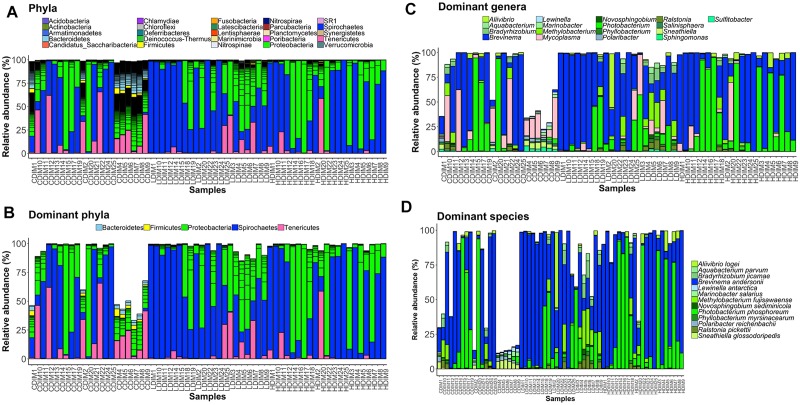
Barplots showing the abundance of the bacterial phyla **(A),** dominant phyla **(B)**, dominant genera **(C)** and dominant species **(D)** in the distal intestinal mucus. The height of each bar segment represents the abundance of individual operational taxonomic units (OTUs) stacked in order from greatest to least and separated by a thin black border line. Color codes: Proteobacteria – green, Spirochaetes – dark blue, Bacteroidetes – light blue, Firmicutes – yellow, Tenericutes – magenta. Sample names starting with CDIM are control distal intestinal mucus, LDIM are AlgOS-L-group distal intestinal mucus and HDIM are AlgOS-H-group distal intestinal mucus.

Proteobacteria and Spirochaetes were more abundant than other taxa, in both content and mucus of AlgOS-fed groups (Figures 3A, 4A). The average relative abundance (%) of the bacterial taxa is given in Table 3.

**TABLE 3:** Average relative abundance (%) of the dominant intestinal bacteria.

**Groups**	**Control**		**AlgOS-L**		**AlgOS-H**	
**Sample type**	**Content**	**Mucus**	**Content**	**Mucus**	**Content**	**Mucus**
**Phyla**						
Proteobacteria	36.00 ± 16.64	38.07 ± 27.80	42.07 ± 22.85	32.13 ± 27.89	35.00 ± 27.13	41.01 ± 39.56
Bacteroidetes	24.15 ± 16.63	10.43 ± 13.85	16.00 ± 17.22	0.35 ± 0.62	4.30 ± 8.20	0
Tenericutes	21.27 ± 28.23	16.54 ± 21.19	12.56 ± 21.79	7.00 ± 12.17	24.22 ± 33.73	5.00 ± 13.31
Firmicutes	11.00 ± 21.94	1.38 ± 1.86	2.00 ± 2.79	0.28 ± 0.54	0.50 ± 0.90	0.12 ± 0.30
Spirochaetes	5.20 ± 8.10	32.30 ± 37.57	26.51 ± 31.74	60.00 ± 34.71	36.00 ± 27.36	54.00 ± 39.45
**Genera**						
*Brevinema*	5.25 ± 8.10	32.29 ± 37.55	26.49 ± 31.73	60.00 ± 34.96	36.00 ± 27.35	54.00 ± 39.43
*Photobacterium*	5.04 ± 8.22	15.02 ± 30.57	20.55 ± 26.11	13.00 ± 18.32	25.54 ± 24.35	35.00 ± 38.28
*Aliivibrio*	0.20 ± 0.49	0.70 ± 1.38	0.69 ± 0.95	3.08 ± 11.71	2.08 ± 5.15	2.12 ± 3.68
*Weissella*	5.00 ± 12.10	0	0	0	0	0
*Sneathiella*	3.13 ± 2.45	1.86 ± 2.46	2.05 ± 2.35	0	0.68 ± 1.36	0
*Polaribacter*	3.16 ± 2.53	1.51 ± 2.17	2.14 ± 2.67	0	0.74 ± 1.44	0
*Lewinella*	4.40 ± 3.71	2.00 ± 2.26	2.33 ± 2.68	0	0.62 ± 1.15	0
*Dokdonia*	1.31 ± 1.15	0.65 ± 0.95	0.98 ± 1.28	0	0.34 ± 0.78	0
*Kordia*	1.46 ± 2.17	0.32 ± 0.56	0.41 ± 0.71	0	0.13 ± 0.37	0
*Marinobater*	1.72 ± 2.08	0.74 ± 1.20	0.85 ± 1.84	0	0.19 ± 0.47	0
*Aquabacterium*	0	0.39 ± 0.83	0	1.24 ± 1.89	0	0.31 ± 0.64
*Bradyrhizobium*	0.17 ± 0.26	1.04 ± 1.93	0.18 ± 0.36	2.80 ± 4.00	0.15 ± 0.40	0.67 ± 1.21
*Methylobacterium*	0.39 ± 0.49	2.11 ± 4.03	0.37 ± 0.71	6.08 ± 8.96	0.28 ± 0.60	1.46 ± 2.86
*Phyllobacterium*	0	0.29 ± 0.52	0	0.88 ± 1.62	0	0.22 ± 0.46
*Ralstonia*	0.10 ± 0.16	0.75 ± 1.48	0.12 ± 0.26	1.89 ± 2.63	0	0.45 ± 0.94
*Novosphingobium*	0	0.15 ± 0.30	0	0.55 ± 0.96	0	0

**The values represent mean ± SD.**

As for the content, Proteobacteria and Spirochaetes were dominant in the AlgOS groups (the former mainly in the AlgOS-L group) compared to the control group (Figure 3B). Tenericutes, Bacteroidetes, and Firmicutes were the other dominant bacterial phyla. Bacteroidetes and Firmicutes in particular were abundant in the DI content of the control fish compared to the AlgOS-fed fish. In the mucus too, AlgOS feeding increased the abundance of the dominant phyla, namely Proteobacteria and Spirochaetes (Figure 4B). Bacteroidetes, Firmicutes, and Tenericutes were also found to be dominant in the mucus, but their abundances were lower in the AlgOS-fed fish compared to the control fish. The average relative abundances (%) of the intestinal bacteria is given in Table 3. Pearson’s Chi-squared test indicated that the proportions of the dominant bacterial phyla in the three groups were significantly different, for both intestinal content (χ^2^ = 5713, *P* < 0.05) and mucus (χ^2^ = 4121, *P* < 0.05). The *P*-values for the Chi-squared test of all the dominant bacterial phyla are given in Supplementary Table S1.

At the genus level, *Brevinema* and *Photobacterium* (*Brevinema andersonii* and *Photobacterium phosphoreum*) were found to be the most dominant ones in the content and mucus of the AlgOS groups compared to the control group (Figures 3C,D, 4C,D). A similar shift in dominance was noted for *Aliivibrio* (*Aliivibrio logei*) too. All the other dominant genera (*Weissella*, *Sneathiella*, *Polaribacter*, *Lewinella*, *Dokdonia* and *Kordia, Marinobacter*) were lower (in the order of 1000–2000) in the content of the AlgOS-fed fish (Figures 3C,D and Supplementary Figures S4A–E). Genera such as *Marinobacter*, *Polaribacter*, *Lewinella* were lower (in the order of 1500–4000) in the DI mucus of the AlgOS-fed salmon (Figures 4C,D and Supplementary Figures S5A–D). *Aquabacterium, Bradyrhizobium*, *Methylobacterium*, *Phyllobacterium*, and *Novosphingobium* (*Aquabacterium parvum, Bradyrhizobium jicamae*, *Methylobacterium fujisawaense*, *Phyllobacterium myrsinaceaum*, *Ralstonia pickettii*, *Novosphingobium sediminicola*) were the abundant (in the order of 2000–10,000) genera in the mucus samples of the AlgOS-L group but reduced in the AlgOS-H group compared to the control group (Figures 4C,D and Supplementary Figures S6A–F). Pearson’s Chi-squared test indicated that the proportions of the dominant bacterial genera in the three groups were significantly different, for both intestinal content (χ^2^ = 5889, *P* < 0.05) and mucus (χ^2^ = 4105, *P* < 0.05). The *P*-values for the Chi-squared test of all the dominant bacterial genera are given in Supplementary Table S1.

### Core and Rare Bacterial Taxa of the Intestinal Microbiota

**FIGURE 5:**
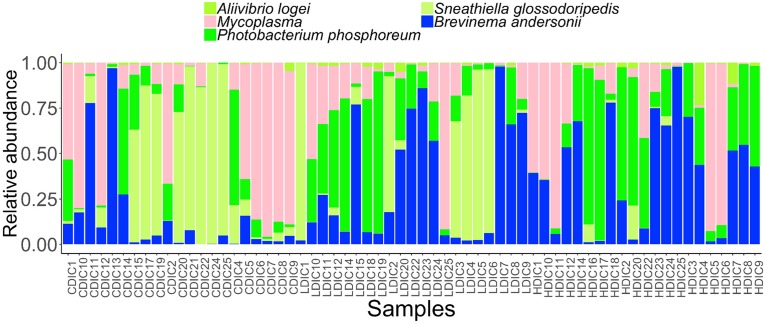
Abundance of the core bacterial taxa in the distal intestinal content of Atlantic salmon from the three study groups. Sample names starting with CDIC are control distal intestinal content, LDIC are AlgOS-L-group distal intestinal content and HDIC are AlgOS-H-group distal intestinal content.

**FIGURE 6:**
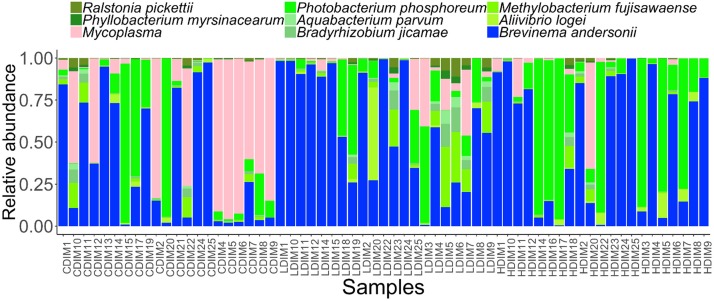
Abundance of the core bacterial taxa in the distal intestinal mucus of Atlantic salmon from the three study groups. Sample names starting with CDIM are control distal intestinal mucus, LDIM are AlgOS-L-group distal intestinal mucus and HDIM are AlgOS-H-group distal intestinal mucus.

We determined the abundance of the common core and rare taxa at prevalence and detection thresholds of 90 and 20%, respectively, to understand if AlgOS supplementation can have an effect on the extant bacterial members and the low abundant rare bacterial members of the intestinal microbiota. The dominant genera in the content and mucus, *Photobacterium* and *Brevinema* (*P. phosphoreum* and *B. andersonii*) were found among the core members (Figures 5, 6). *Aliivibrio, Sneathiella* (*A. logei, S. glossodoripedis*) and *Mycoplasma* were also shared core taxa of the content. The common core taxa in the mucus included the aforementioned core taxa of the content (except *S. glossodoripedis)* and other genera such as *Phyllobacterium*, *Aquabacterium*, *Ralstonia*, *Methylobacterium*, *Bradyrhizobium* (*P. myrsinacearum*, *A. parvum*, *R. pickettii*, *M. fujisawaense*, and *B. jicamae*). The DPCoA plot showed differential clustering of the core members of the AlgOS and control groups (content: F-statistic: 3.715, *R*^2^ = 0.128, *P* < 0.001, mucus: F-statistic: 4.072, *R*^2^ = 0.137 1.0, *P* < 0.01 − Supplementary Figures S7A,B).

### The Significantly Different and Relevant Bacterial Communities of the Intestinal Microbiota

In the DI content, AlgOS-fed fish had certain groups of significantly (*P* < 0.05) abundant bacteria compared to the control fish. Firmicutes, Spirochaetes, and Proteobacteria were the abundant phyla (Supplementary Figure S8A), and Spirochaetia and Gammaproteobacteria were the significantly abundant classes (Supplementary Figure S8B). Spirochaetales, Vibrionales (order), Vibrionaceae, Brevinemataceae (family), *Brevinema*, *Photobacterium*, and *Aliivibrio* were the significantly abundant bacteria (Supplementary Figures S8C–E, respectively).

In the DI mucus, the phyla Actinobacteria and Proteobacteria were significantly reduced in the AlgOS-L group (Supplementary Figure S9A). In addition, Betaproteobacteria was the significantly abundant class in the AlgOS-L group (Supplementary Figure S9B). Burkholderiales, Alcaligenaceae, Sphingobacteriaceae, Burkholderiaceae, Microbacteriaceae, *Achromobacter*, *Aquabacterium*, *Novosphingobium*, and *Micrococcus* were also significantly abundant in the AlgOS-L group (Supplementary Figures S9C–E).

Here we report the species that were significantly different in the AlgOS-fed fish. *P. phosphoreum, A. logei*, and *B. andersonii* were significantly abundant in the content of the AlgOS-fed fish (Supplementary Figure S8F). *Achromobacter insolitus, Aquabacterium parvum, N. sediminicola*, and *Microbacterium ginsengiterrae* were significantly abundant in the mucus of the AlgOS-L group compared to the control group (Supplementary Figure S9F).

We have also identified the relevant bacterial communities employing Boruta analysis, which detected 6 and 4 relevant OTUs in content and mucus samples, respectively. These OTUs discriminate the three study groups, i.e., OTUs with higher abundance in one study group (Supplementary Figures S10A–E). In the DI content, Gammaproteobacteria, *Acetanaerobacterium*, Alteromonadaceae, Desulfuromonadales, and few taxa belonging to Bacteriodetes (*Psychroserpens jangbogonensis*, Supplementary Figure S10A, and Winogradskyella) were found to be relevant for the discrimination. An OTU of *Acetanaerobacterium* (*A. elongatum*, belonging to Firmicutes; Supplementary Figure S10B) and Gammaproteobacteria were found to be abundant in the AlgOS-H and AlgOS-L groups, respectively compared to control group. In DI mucus, genera such as *Phyllobacterium*, *Undibacterium* and *Microbacterium* (*Phyllobacterium myrsinacearum, Undibacterium oligocarboniphilum*, and *M. ginsengiterrae*) were found to be abundant in the AlgOS-L group (Supplementary Figures S10C–E).

### Indication of Butyrate-Producing and Carbohydrate-Degrading Ability of the Significantly Abundant and Relevant Intestinal Bacteria

The gene clusters associated with butyrate production—from substrates such as 4-aminobutyrate and pyruvate—were present in *A. insolitus*, *A. parvum*, and *P. myrsinacearum*. *A. logei* is capable of producing butyrate from glutarate and *B. andersonii* has the genes for the 4-aminobutyrate pathway that is necessary for butyrate production. *A. insolitus* and *A. parvum* can produce butyrate via the pyruvate pathway. *P. myrsinacearum* can use both pyruvate and 4-aminobutyrate pathways to produce butyrate (Table 1). CAZyme families are present in the mentioned genomes (Table 2); glycoside hydrolases and polysaccharide lyases.

## Discussion

Prebiotics are intended to selectively target host microorganisms that can ferment the indigestible carbohydrate and stimulate the growth of specific bacteria to produce bioactive metabolites (Gibson et al., 2017). The beneficial bacteria and their metabolites such as SCFAs are known to provide health benefits to the hosts (Gibson et al., 2017). SCFAs can affect the pH of the colonic microenvironment, and this in turn can influence the community composition (Arnoldini et al., 2018). Furthermore, the gut microbiota can be reshaped by fine-tuning the carbohydrate food components; to improve host health status and tackle diseases, as in the case of mice fed a marine polysaccharide (Shepherd et al., 2018). In addition, it has been shown that caloric restriction is associated with the dominance of a particular beneficial bacterial type in rat models (Fraumene et al., 2018). In the present study, we investigated the effect of *Laminaria* sp.-derived AlgOS on the diversity and composition of bacterial communities in the intestine of Atlantic salmon. We analyzed the DI content and mucus separately to understand the differences in the microbiota associated with them. In addition, the bacterial composition of the environmental samples were found to be significantly different from the respective fish-associated microbial communities. Other studies have also reported similar differences in the host and environmental bacteria (Lyons et al., 2017).

Our results revealed that AlgOS supplementation causes an overall reduction in bacterial diversity of the DI bacterial community of the fish fed 2.5% AlgOS (AlgOS-H group) compared to the control fish. However, 0.5% AlgOS supplementation (AlgOS-L group) in feed effected similar changes without lowering the bacterial diversity. In this fish group, the phyla Proteobacteria and Spirochaetes were dominant, in both DI content and mucus. Certain species of Proteobacteria (dominant), Spirochaetes (dominant) and Actinobacteria (low abundance) were relatively abundant/significantly abundant bacteria in the 0.5% AlgOS-fed fish.

### AlgOS Reduces the Diversity of the Intestinal Bacteria

Previous studies have shown that oligosaccharides like GOS and inulin reduced the bacterial diversity in mouse fecal samples (Cheng et al., 2017). Similarly, pectic oligosaccharides also decreased the microbial diversity and richness of cecal microbiota in mice (Bindels et al., 2015). Furthermore, simplified cecal microbiota was a characteristic of rats fed alginate compared with those fed a control diet (An et al., 2013). In contrast to our observations, an increase in the intestinal bacterial diversity under the influence of oligosaccharide supplementation has been previously reported in gilthead sea bream (Dimitroglou et al., 2010) and rats (Ou et al., 2016). On the other hand, a prebiotic blend (FOS + GOS + inulin + anthocyanins) did not alter the diversity of the gut microbiota of mice (Chen et al., 2017).

Piazzon et al. (2017) have shown that the intestinal bacteria of gilthead sea bream (*Sparus aurata*) fed more plant-derived ingredients had apparently lower Shannon index. The alpha diversity of the intestinal bacterial of largemouth bronze gudgeon (*Coreius guichenoti*) suffering from furunculosis was significantly lower compared to healthy fish (Li et al., 2016). The reduction in microbial diversity of the intestinal microbiota of AlgOS-H salmon group is intriguing and the loss in the number of beneficial bacteria has to be verified further. It should be noted that the intestinal microbial diversity of the AlgOS-L fish group was not impacted significantly. Ecological stability in a gut environment is linked to high microbial diversity, and preservation of functions of beneficial symbionts (Chassard et al., 2008). However, high bacterial diversity and increase in cooperating microbes could jeopardize the ecological stability (Coyte et al., 2015). Hence a decrease in bacterial diversity does not always point to an unstable ecosystem. In addition, it is known that the luminal flow rate and muscle contraction-induced mixing strength can affect the density and stability of human colonic microbiota (Arnoldini et al., 2018). Furthermore, decrease in intestinal bacterial diversity, shifts in bacterial compositions and disruptions of community functions are associated with ill-health (Li et al., 2016). Future studies should reveal the competing and cooperating communities in the AlgOS-fed fish.

### AlgOS Facilitated the Dominance of Proteobacteria, Spirochaetes, and Actinobacteria

The significantly abundant and the relevant species indicate that AlgOS stimulated the growth of certain bacteria belonging to Proteobacteria, Spirochaetes and Actinobacteria, especially in AlgOS-L fish. On the other hand, AlgOS reduced the abundance of certain Firmicutes and Bacteroidetes.

Members of the phylum Bacteroidetes are prominent among the gut microbiota – they can be pathogens and have the capacity to degrade polysaccharides (Thomas et al., 2011). The metabolic functions of the Bacteroidetes that were reduced in the ALgOS-fed fish are still unknown. Earlier studies have shown that oligosaccharide can reduce the abundance of Firmicutes in the gut microbiota of mice (Petersen et al., 2010) and humans (Vigsnaes et al., 2011), as noted in the present study. Studies have reported that Firmicutes and Bacteriodetes are dominant in the fecal samples of Atlantic salmon during early summer, but later on lose their dominance to Proteobacteria (Zarkasi et al., 2014). Thus, prebiotics are likely to affect the dominant phyla of mammals and fish.

Previous studies have also demonstrated that Proteobacteria is the most abundant phylum in many marine and freshwater fishes (Roeselers et al., 2011; Li et al., 2015; Liu et al., 2016; Lokesh and Kiron, 2016), and it is also known to dominate the gut microbiota of Atlantic salmon (Gajardo et al., 2016; Gupta et al., 2019). Therefore, it is not surprising to find Proteobacteria as one of the most abundant and dominant bacterial phyla. Studies have reported that Proteobacteria are involved in metabolic pathway modules that participate in carbon and nitrogen fixation and in the stress response regulatory system (Vikram et al., 2016). Proteobacteria may also contribute to the digestive process in fish (Romero et al., 2014). *P. phosphoreum* belonging to the class *Gammaproteobacteria* is a known gut symbiont of marine fish, and this bacterium is capable of chitin digestion and uses luciferase to reoxidize reduced coenzymes and other molecules for metabolism (Nealson and Hastings, 1979). Although this species was significantly abundant in the content of the AlgOS-fed fish, we did not find a corresponding abundance in the mucus. Soybean meal can decrease the abundance of *P. phosphoreum* in the DI of Atlantic salmon (Desai et al., 2012). However, we observed an increase in abundance of this bacterium and another member of Proteobacteria (*A. logei*) as a result of AlgOS feeding.

In the mucus of AlgOS-L fish, few members of Proteobacteria including *A. parvum, B. jicamae*, and *M. fujisawaense* (Supplementary Figures S6A–C) were significantly abundant. *A. parvum* is known as a nitrate-dependent Fe(II)-oxidizing bacterium (Zhang et al., 2016). In the genus *Bradyrhizobium*, many bacteria are known to fix nitrogen (Peix et al., 2015), but currently, no information is available for *B. jicamae.* Hsiang-Yi et al. (2018) have reported the presence of *B. jicamae* in the intestinal microbiota of anguillid eel species. Although *Methylobacterium* species are methylotrophs and they are described as agents of contamination and infections in humans (Lai et al., 2011), details of *M. fujisawaense* are not yet reported.

The discriminatory OTUs, revealed through Boruta analysis, indicated that Gammaproteobacteria and Alteromonadaceae were abundant in the content of the AlgOS-L group. Likewise, the relevant OTUs in the mucus were abundant in the AlgOS-L group, and most of them were Proteobacteria (*P. myrsinacearum*, *U. oligocarboniphilum* (Supplementary Figures S10C,D). *P. myrsinacearum* belongs to Alphaproteobacteria; this bacterium that is associated with macroalgae is a nitrogen fixer (Gonzalez-Bashan et al., 2000). Its abundance increased in the AlgOS-L group but decreased in the AlgOS-H group, compared to the control fish. *P. myrsinacearum* has been reported in intestinal mucosa of grass carp, *Ctenopharyngodon idellus* (Huan et al., 2015). The functional relevance of *U. oligocarboniphilum* (Betaproteobacteria) for the host is not yet described.

Spirochaetes is the second most abundant and dominant phylum in the AlgOS-fed fish. *B. andersonii* was found to be the dominant species; it was also one of the core bacterial members of the DI of Atlantic salmon. Tapia-Paniagua et al. (2014) has reported *B. andersonii* in the intestinal microbiota of flat fish (*Solea senegalensis*). While Spirochaetes include species that cause disease in vertebrates, they are also known as abundant endosymbionts and lignocellulose digesters and nitrogen fixation helpers in termite guts (Kudo, 2009). However, functional information of this bacteria in the gut of fish is not yet reported. *B. andersonii* was significantly higher in the content of Atlantic salmon that consumed AlgOS-containing feeds. Our *in silico* analyses indicate that *B. andersonii* has genes that are necessary for butyrate production. In addition to the dominant Proteobacteria, some dominant/rare species of Actinobacteria, were relatively abundant/significantly abundant in the AlgOS-L fish. The abundance of *M. ginsengiterrae*, a β-glucosidase-producing bacterium (Kim et al., 2010) which belongs to Actinobacteria, increased in the AlgOS-L group. However, their functional role in the gut of the fish needs to be elucidated.

### Low-AlgOS Stimulated the Abundance of Bacteria With Possible Carbohydrate-Degrading and Butyrate-Producing Ability

Butyrate production occurs via pyruvate by breakdown of complex polysaccharides, or via amino acids which serves as substrates for lysine, glutarate and 4-aminobutyrate pathways. However, all the pathways have a common step where crotonyl-CoA is transformed to butyryl-CoA (Vital et al., 2014). Pathway analysis suggested that the intestinal bacteria in our study have gene clusters for pyruvate and acetyl-CoA pathway; most of the reported butyrate producers are known to synthesize butyrate via pyruvate pathway (Vital et al., 2014; Anand et al., 2016). Intriguingly, the genomes we examined have CAZymes − GHs and PLs, which are necessary to break down dietary carbohydrates (Lombard et al., 2013). Particularly the families GH3 and PL7 are known to be associated with algal polysaccharide utilization (Kabisch et al., 2014), and GH5 is linked to cellulose degradation (Park and Kong, 2018). The glucoside hydrolases, GH1 and GH3, are known to produce glucose from cellobiose (Park and Kong, 2018). Furthermore, among the listed bacteria in our study, *P. myrsinacearum* has the genes for both pyruvate and 4-aminobutyrate pathways. Vital et al. (2014) have reported that members of phyla, other than Firmicutes, especially Actinobacteria, Bacteroidetes, Fusobacteria, Proteobacteria, and Spirochaetes are potential butyrate producers.

Mountfort et al. (2002) have measured the SCFAs in the hindgut of three marine herbivorous fishes and related the production to the gut microbiota; the production rate of one of the predominant SCFAs i.e., acetate in three herbivorous fin fishes and terrestrial vertebrates suggests that body temperatures do not affect the fermentation systems of the metabolic groups of their gut bacteria. Kihara (2008) showed that the increased production of SCFA in the hindgut of red seabream administered with oligosaccharide lactosucrose, although carnivorous fish are known to have lower fermentation rates. In wild carnivorous freshwater fishes, *Cetobacterium* and *Halomonas* are highly abundant, while their herbivorous counterparts were enriched with *Citrobacter* and *Leptotrichia* (Liu et al., 2016). Certain bacteria present in herbivorous fish (e.g., Vibrionales, Clostridiales) (Sullam et al., 2012) are found in the DI of Atlantic salmon also (Gupta et al., 2019). Furthermore, morphology of the digestive system will not affect the fermentation reactions in the hindgut of fishes (Mountfort et al., 2002). However, efficient fermentation of polysaccharides in the gut ecosystem requires an optimum number of certain functional bacterial groups (Chassard et al., 2008). Therefore, *in silico* and culture-based studies can provide knowledge about the contribution of fish intestinal bacteria to butyrate production. In the present study, *A. logei*, *A. insolitus*, *A. parvum*, and *P. myrsinacearum* that were found to be abundant in the content and mucus of the AlgOS-L group had genes linked to CAZymes and genes associated with butyrate production. The role of butyrate in maintaining the host GI health has been well documented in humans and other animals (Segain et al., 2000; Fukumoto et al., 2003). Studies have shown the potential impact of dietary butyrate in fish: in carp (*Cyprinus carpio*) it improves growth (Liu et al., 2014), in gilthead sea bream (*Sparus aurata*) it might provide energy to the enteric cells and promote absorption of essential amino-acids (Robles et al., 2013), and help restore the intestinal health (Estensoro et al., 2016). Culture-dependent studies are required to ascertain the ability of the above-mentioned high abundant bacteria in AlgOS-L group in stimulating the production of butyrate in Atlantic salmon.

## Conclusion

This comprehensive characterization has revealed the effects of dietary supplementation of the *Laminaria* sp.-derived AlgOS on the intestinal bacterial communities of Atlantic salmon. The dietary supplementation of the Laminaria sp.-derived AlgOS (2.5%) reduced the overall intestinal bacterial diversity. The low (0.5%) AlgOS did not lower the diversity, but facilitated the dominance of a few members of Proteobacteria (viz. *P. phosphoreum*, *A. logei, A. parvum*, *A. insolitus*), Spirochaetes (*B. andersonii*), and Actinobacteria (*M. ginsengiterrae*) in the content or mucus of the fish fed the low level of the AlgOS. Among these bacteria *A. logei, A. parvum*, *B. andersonii, A. insolitus* have the capacity to degrade carbohydrates and produce butyrate, suggesting that 0.5% AlgOS supplemented feed additive may be good for the fish. Although the *in silico* findings have revealed the presence of CAZyme and butyrate genes in the genome of selected abundant bacteria associated with the 0.5% AlgOS fed fish, future research should confirm the abundance of short chain fatty acid production after feeding the oligoalginate. This information will be useful for studies that explore the metabolic potential of oligosaccharide-stimulated gut bacteria and their effect on the host.

## Data Availability

The datasets generated for this study can be found in European Nucleotide Archive (ENA), PRJEB27188.

## Author Contributions

VK, MS, and JF procured the funding for the study. VK, MS, RP, JF, and SG designed the study. RP provided the AlgOS. VK, MS, and SG conducted the feeding experiment. SG performed the microbiota studies including the laboratory work and wrote the manuscript with the guidance of VK. SG, JL, PS, VK, and JF analyzed the data. YA performed the *in silico* butyrate pathway analysis. All authors read, revised, and approved the manuscript.

## Conflict of Interest Statement

The authors declare that the research was conducted in the absence of any commercial or financial relationships that could be construed as a potential conflict of interest.

## Supplementary Material

FIGURE S1 Boxplots showing the Faith’s phylogenetic diversity of the bacterial communities of distal intestinal content **(A)** and mucus **(B)**. Different letters indicate statistically significant differences (*P* < 0.05) between the study groups.

FIGURE S2 Double principal coordinate analysis plot showing the beta diversity of the tank water bacterial communities.

FIGURE S3 Double principal coordinate analysis plots showing the beta diversity of the bacterial communities. Tank biofilm bacteria **(A)**, control distal intestinal content and biofilm bacteria: F-statistic = 2.0828, *R*^2^ = 0.0798, *P* > 0.13 **(B)**, AlgOS-L distal intestinal content and biofilm bacteria: F-statistic = 2.3027, *R*^2^ = 0.0875, *P* > 0.86 **(C)**, AlgOS-H distal intestinal content and biofilm bacteria: F-statistic = 3.7455, *R*^2^ = 0.1349, *P* > 0.027 **(D)**, control distal intestinal mucus and biofilm bacteria: F-statistic = 2.3912, *R*^2^ = 0.906, *P* > 0.074 **(E)**, AlgOS-L distal intestinal mucus and biofilm bacteria: F-statistic = 9.9021, *R*^2^ = 0.2920, *P* > 0.002 **(F)**, AlgOS-H distal intestinal mucus and biofilm bacteria: F-statistic = 6.2846, *R*^2^ = 0.2075, *P* > 0.012 **(G)**.

FIGURE S4 Barplots showing the abundance of bacterial species in the distal intestinal content. *Weissella cibaria* **(A)**, *Sneathiella glossodoripedis* **(B)**, *Polaribacter reichenbachii* **(C)**, *Lewinella antarctica* **(D)**, *Kordia antarctica* **(E)**.

FIGURE S5 Barplots showing the abundance of bacterial species in the distal intestinal mucus. *Marinobacter salarius* **(A)**, *Sneathiella glossodoripedis* **(B)**, *Polaribacter reichenbachii* **(C)**, *Lewinella antarctica* **(D)**.

FIGURE S6 Barplots showing the abundance of bacterial species in the distal intestinal mucus of AlgOS-L group. *Aquabacterium parvum* **(A)**, *Bradyrhizobium jicamae* **(B)**, *Methylobacterium fujisawaense* **(C)**, *Phyllobacterium myrsinacearum* **(D)**, *Ralstonia pickettii* **(E)**, *Novosphingobium sediminicola* **(F)**.

FIGURE S7 Double principal coordinate analysis plots showing the beta diversity of the core taxa of the distal intestinal content **(A)** and the beta diversity of the core taxa of the distal intestinal mucus **(B)**.

FIGURE S8 Polar plots showing the differences in abundances of significantly different intestinal content bacteria, at different taxonomic levels. Phylum **(A)**, class **(B)**, order **(C)**, family **(D)**, genus **(E)**, and species **(F)**.

FIGURE S9 Polar plots showing the differences in abundances of significantly different intestinal mucus bacteria, at different taxonomic levels. Phylum **(A)**, class **(B)**, order **(C)**, family **(D)**, genus **(E)**, and species **(F)**.

FIGURE S10 Barplots showing the abundance of relevant bacterial taxa in the distal intestinal content and mucus of the AlgOS-fed groups. *Psychroserpens jangbogonensis* **(A)**, *Acetanaerobacterium elongatum* **(B)**, *Phyllobacterium myrsinacearum* **(C)**, *Undibacterium oligocarboniphilum* **(D)** and *Microbacterium ginsengiterrae* **(E)**.

TABLE S1 Contingency table for the OTU counts in the content and mucus.
