# Supplementary material for: Macroalga-Derived Alginate Oligosaccharide Alters Intestinal Bacteria of Atlantic Salmon
Source: Front Microbiol. 2019 Sep 13;10:2037. doi: 10.3389/fmicb.2019.02037 (PMC6753961; doi:10.3389/fmicb.2019.02037)

Supplementary Figure 1A

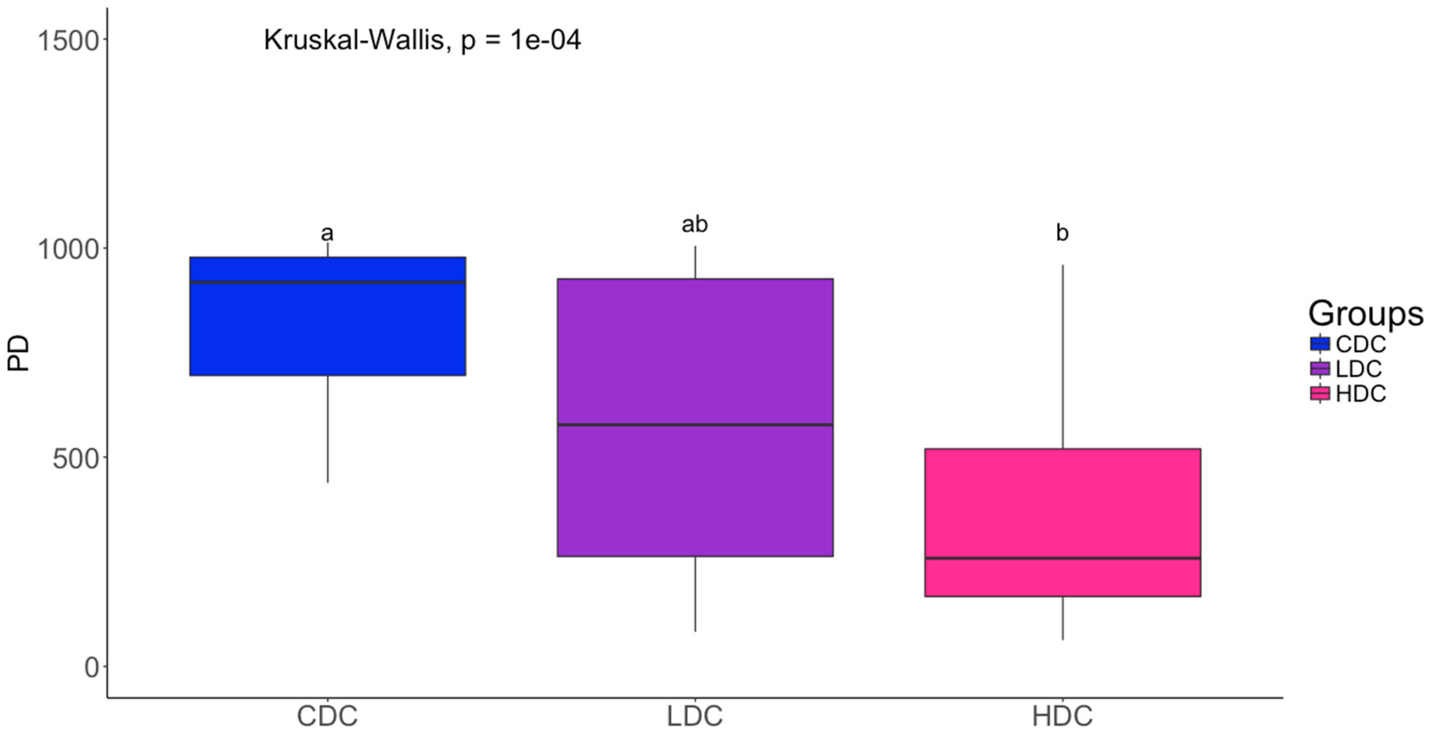

Supplementary Figure 1B

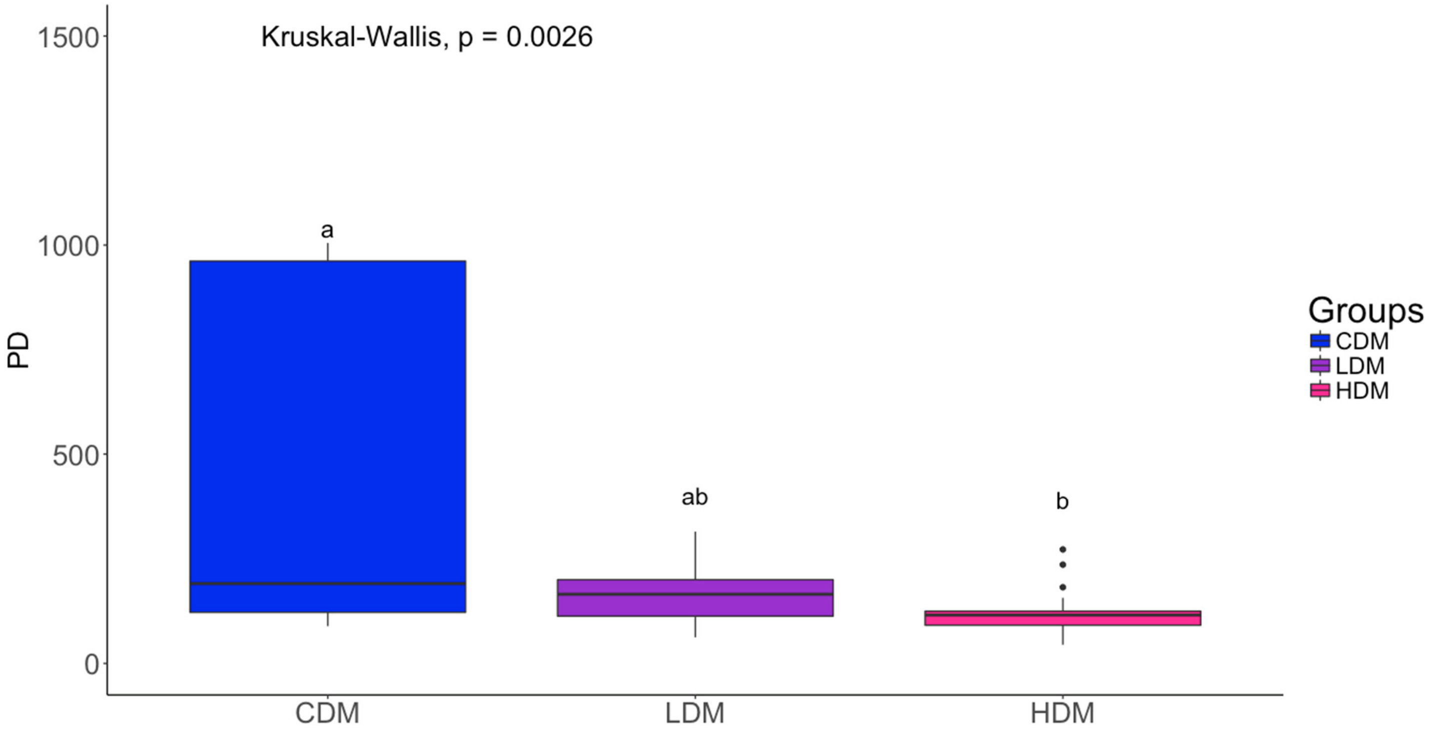

Supplementary Figure 2

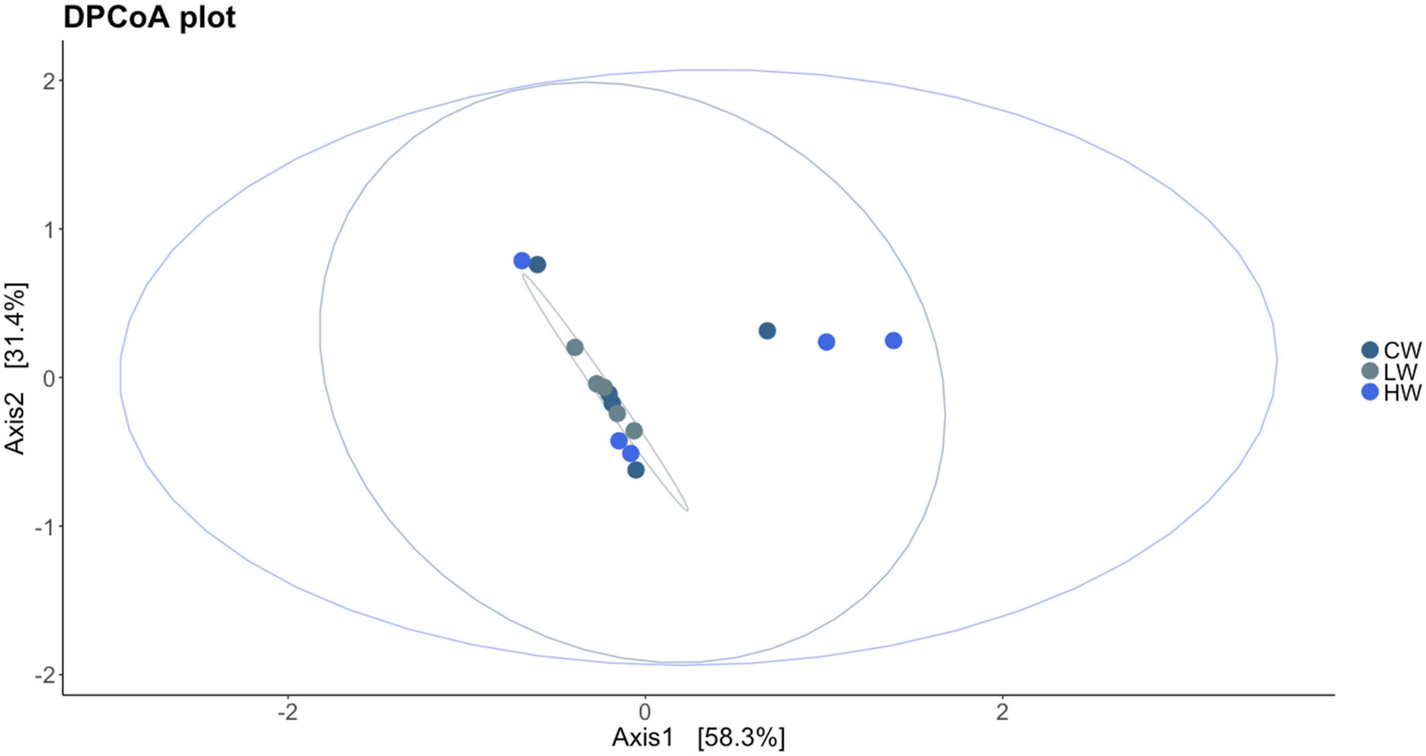

Supplementary Figure 3A

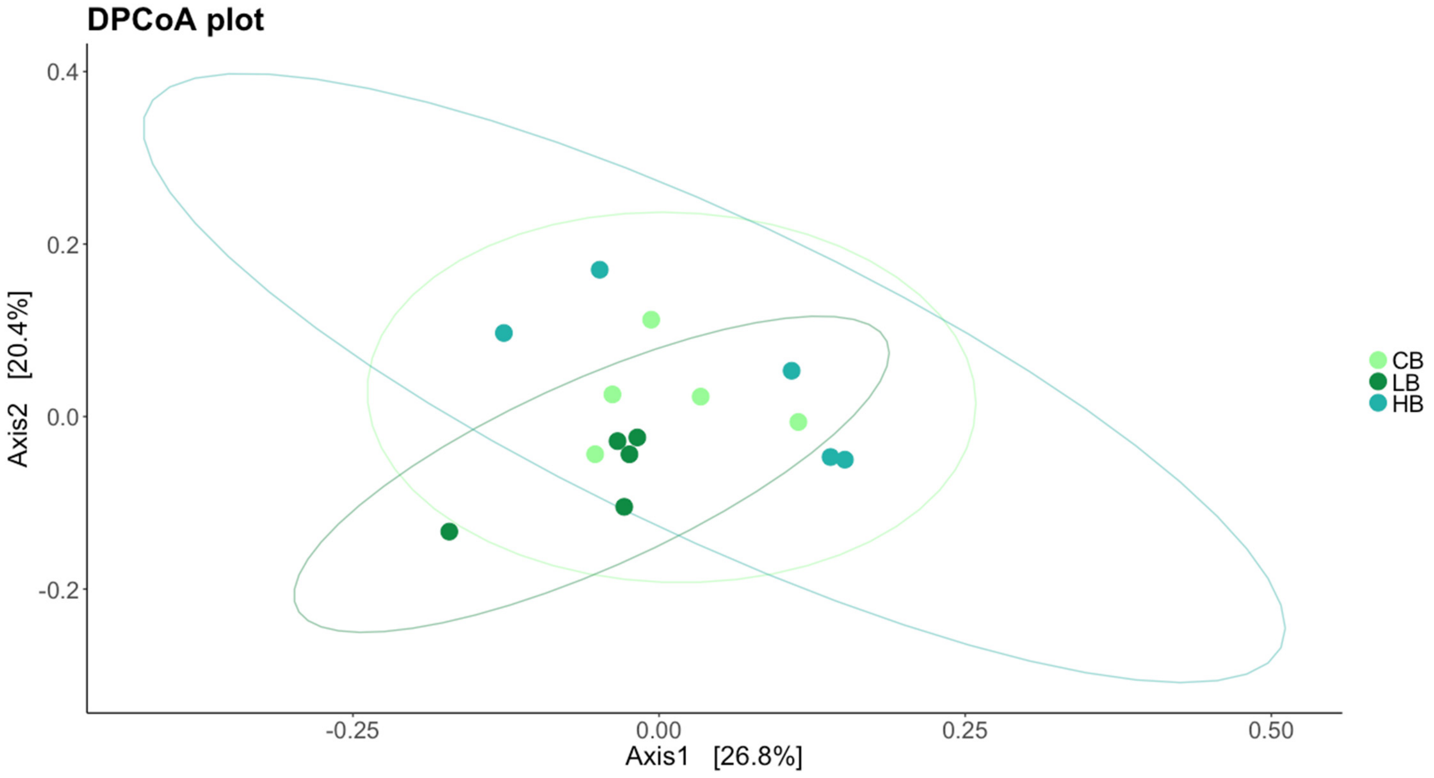

Supplementary Figure 3B

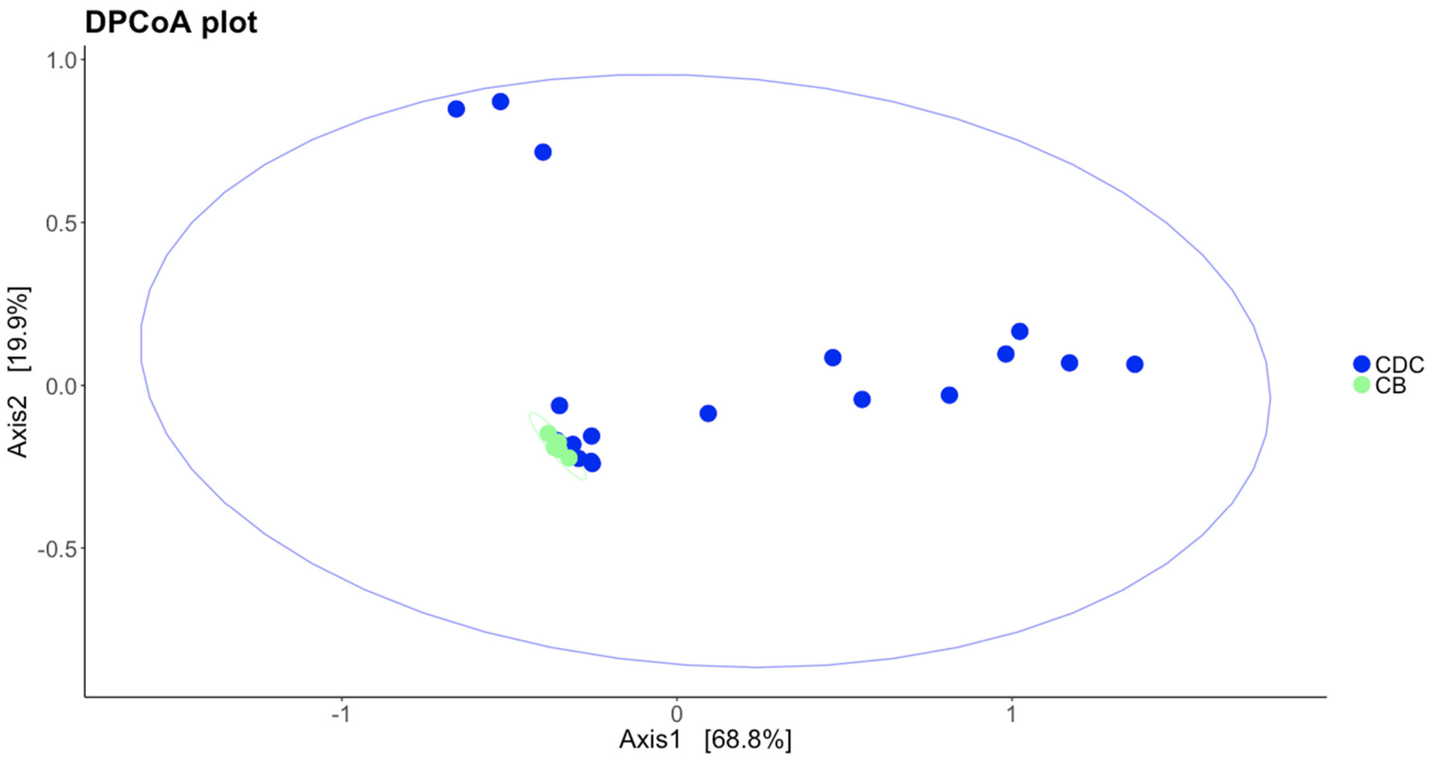

Supplementary Figure 3C

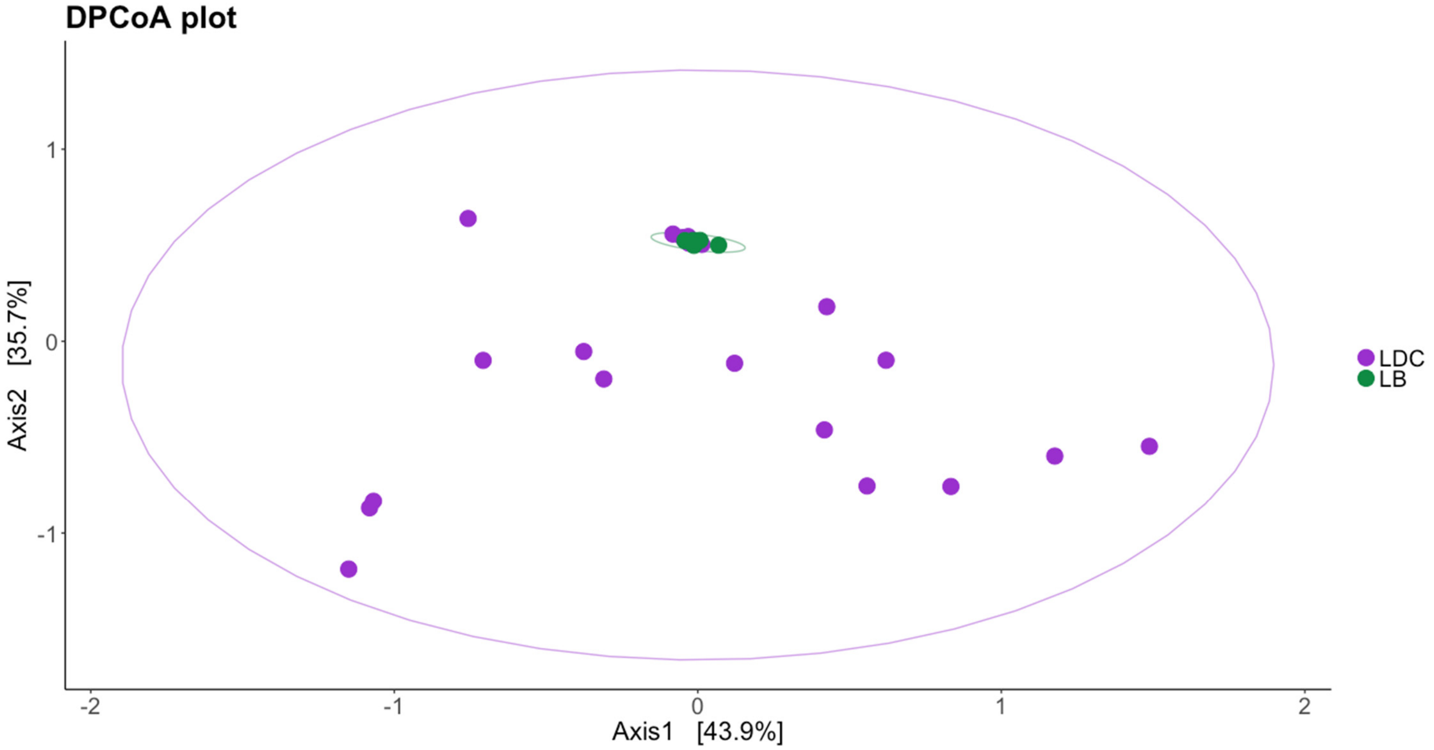

Supplementary Figure 3D

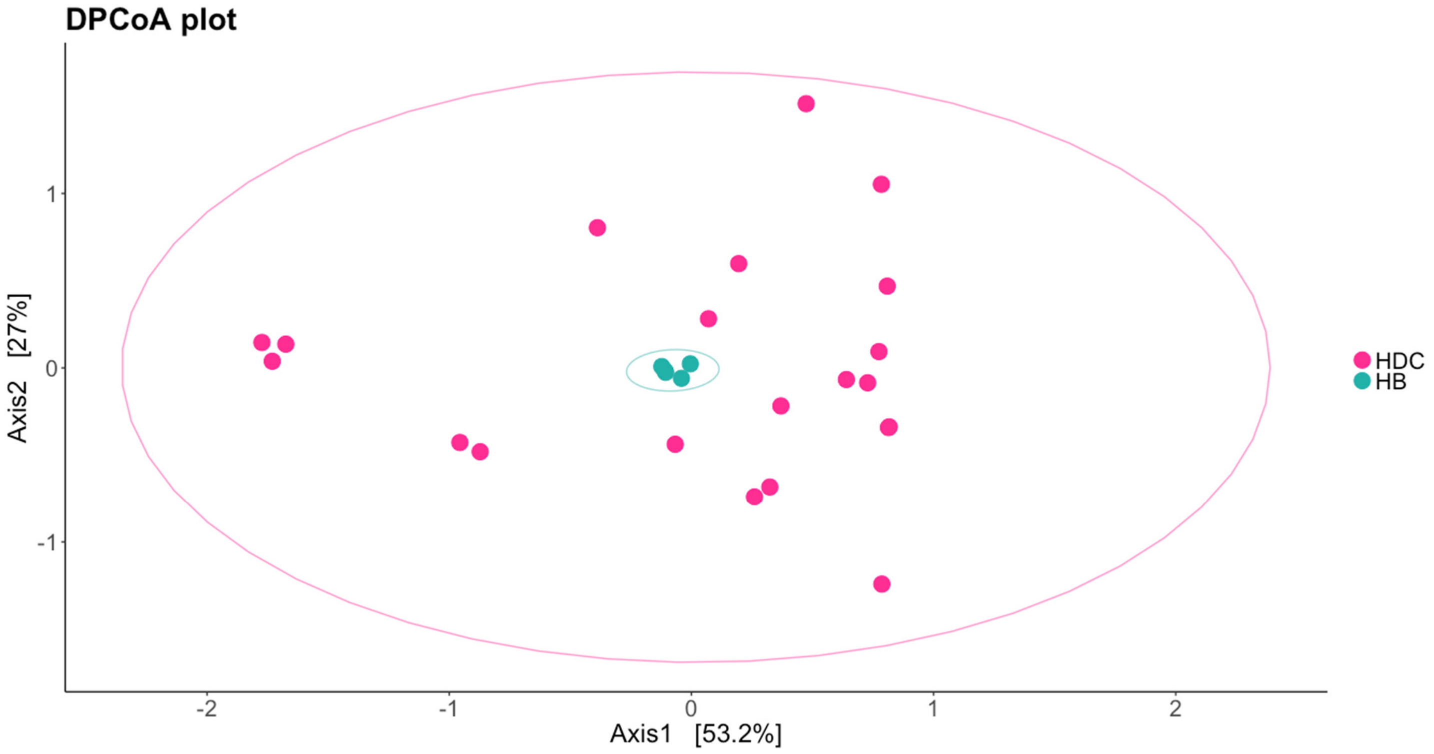

Supplementary Figure 3E

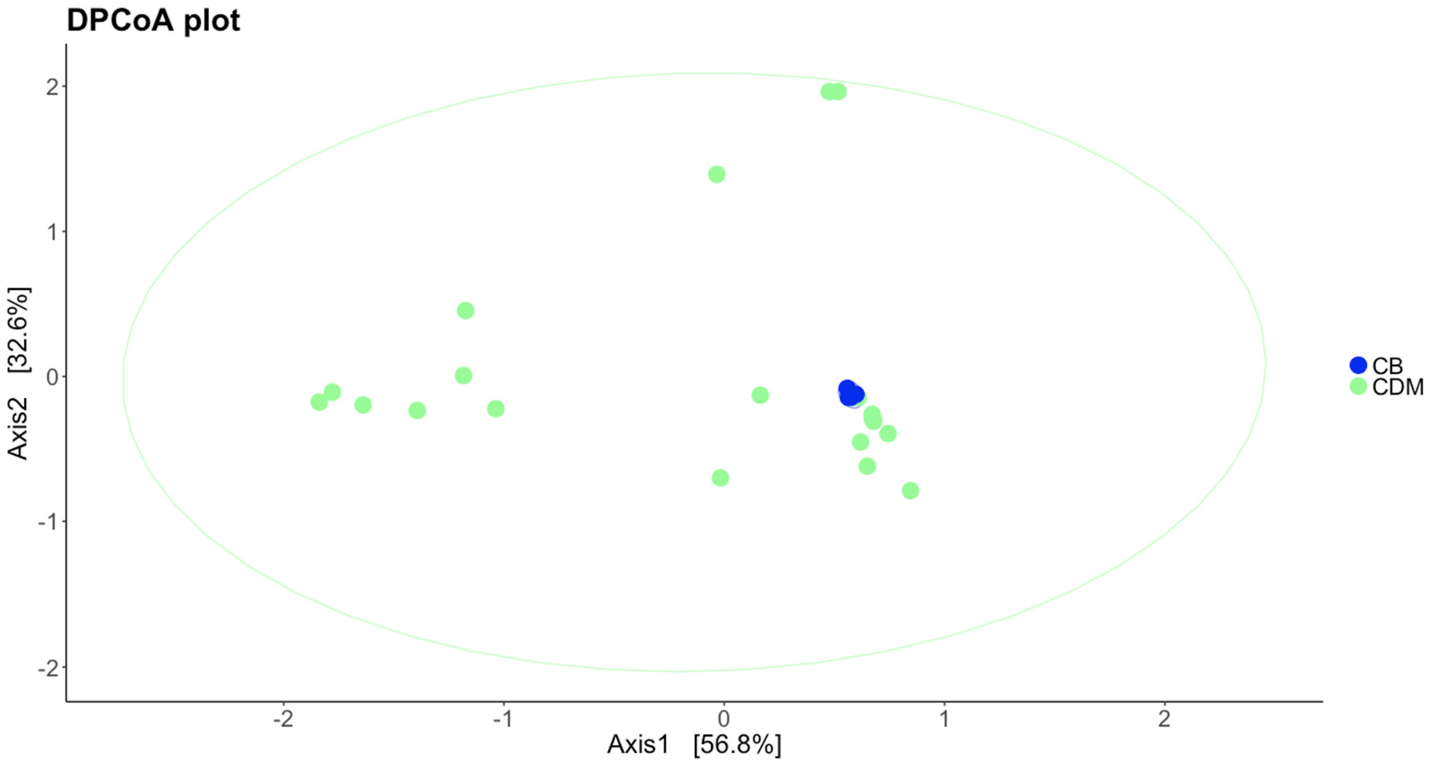

Supplementary Figure 3F

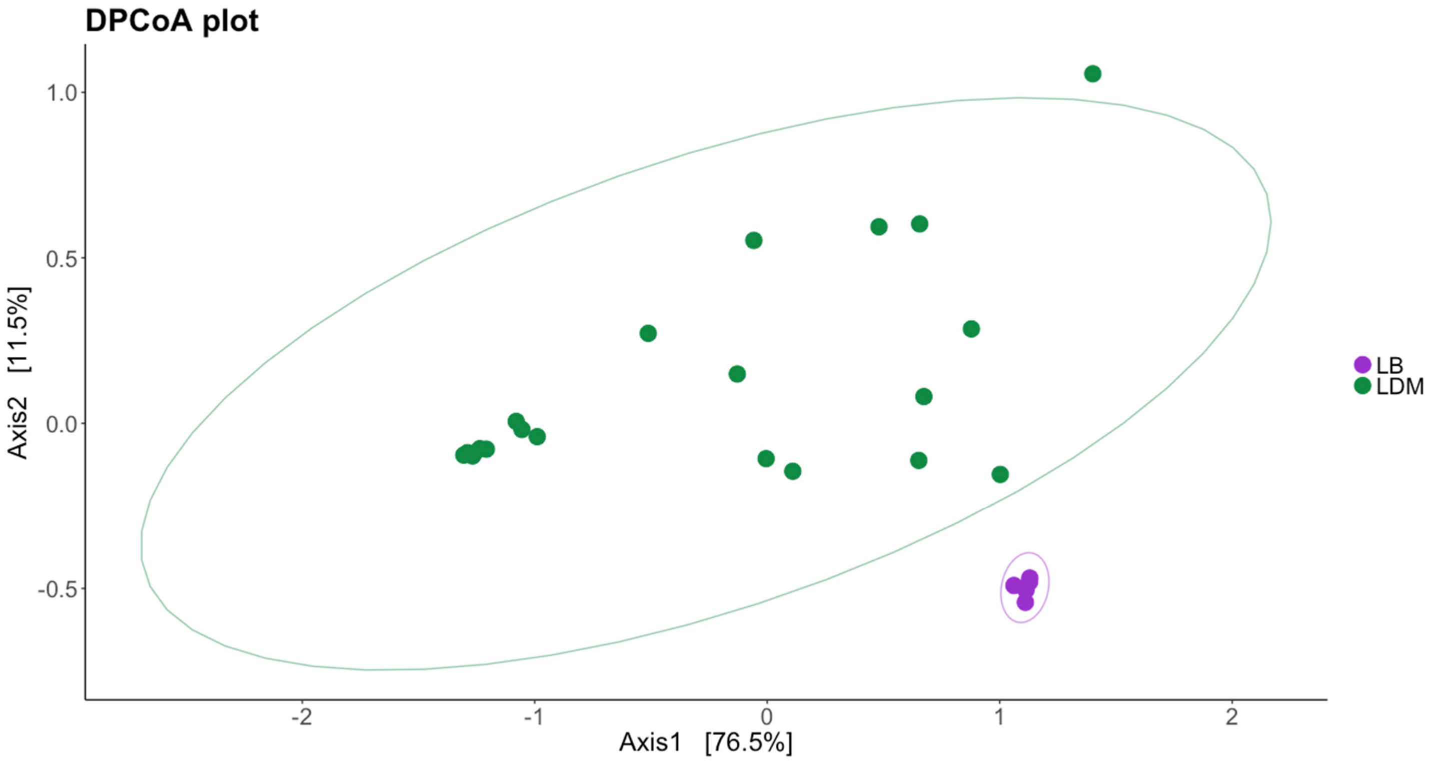

Supplementary Figure 3G

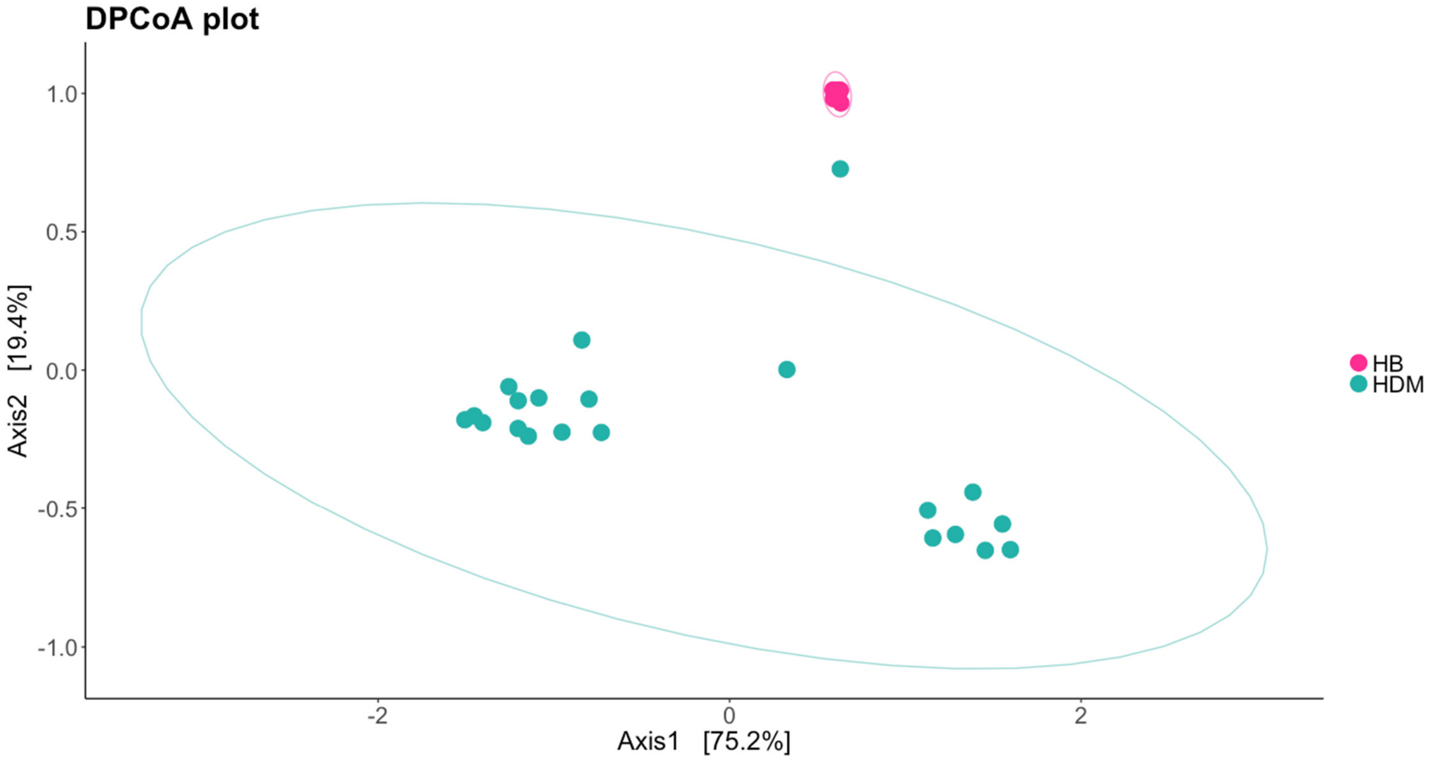

Supplementary Figure 4A

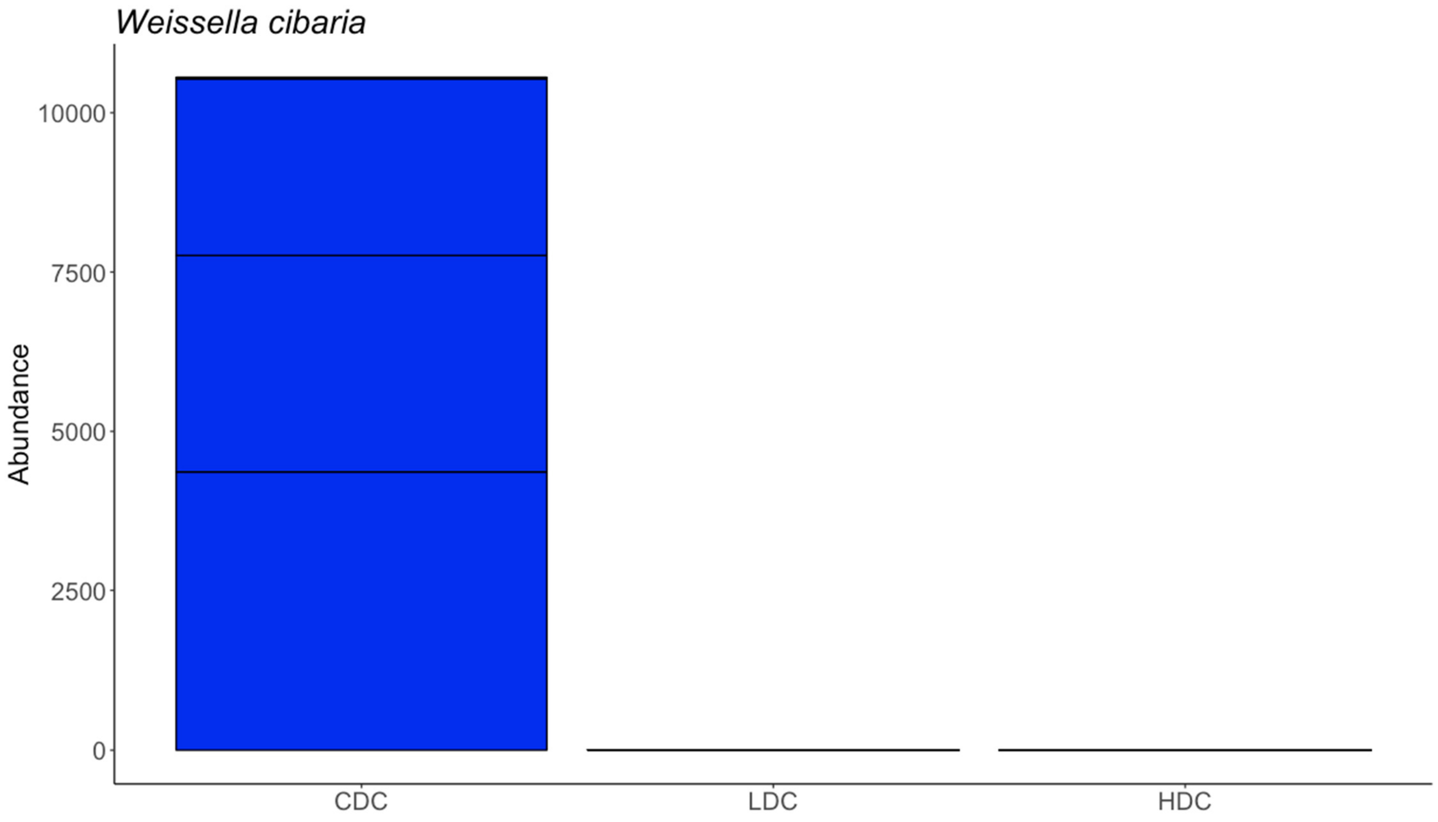

Supplementary Figure 4B

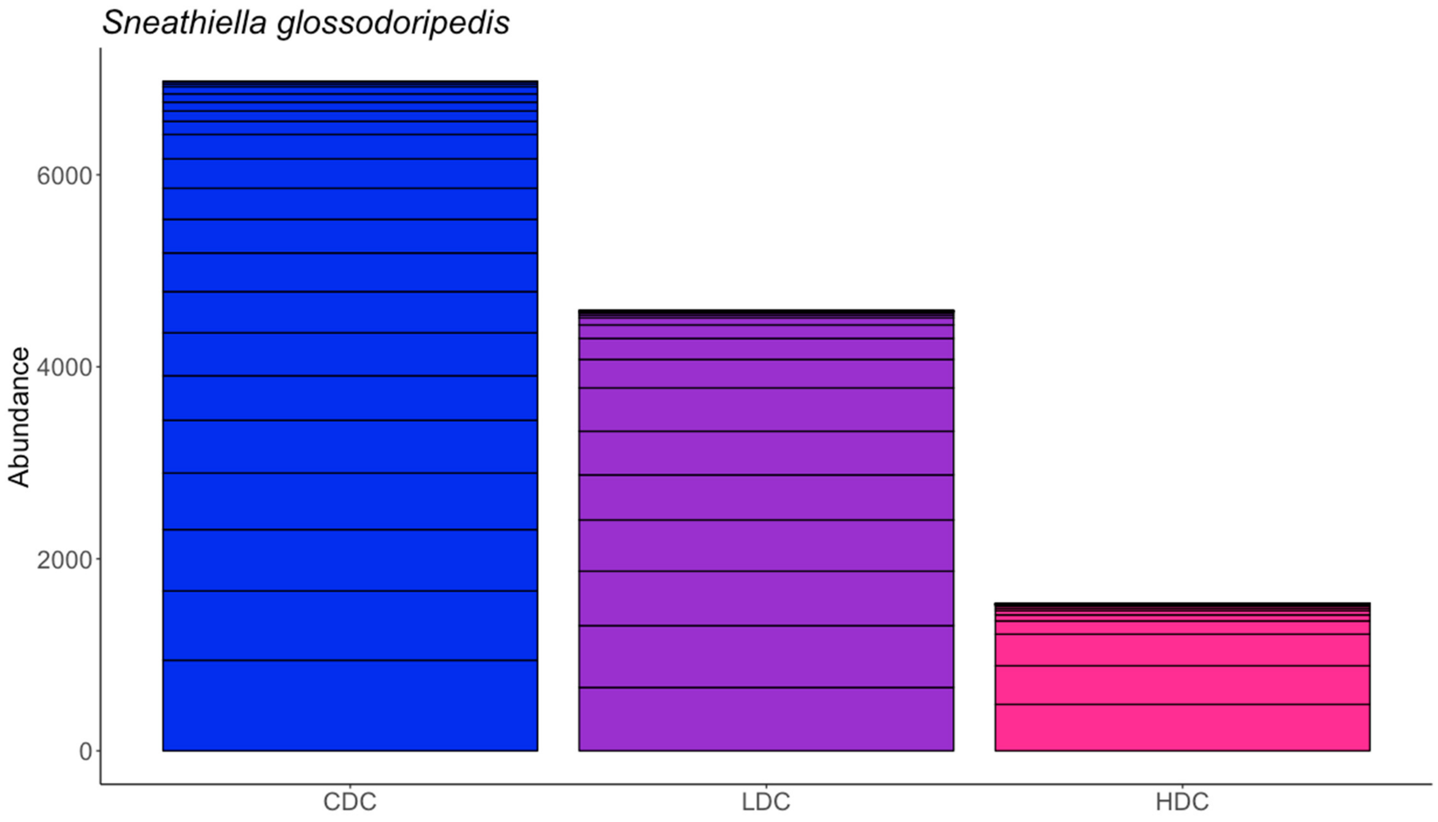

Supplementary Figure 4C

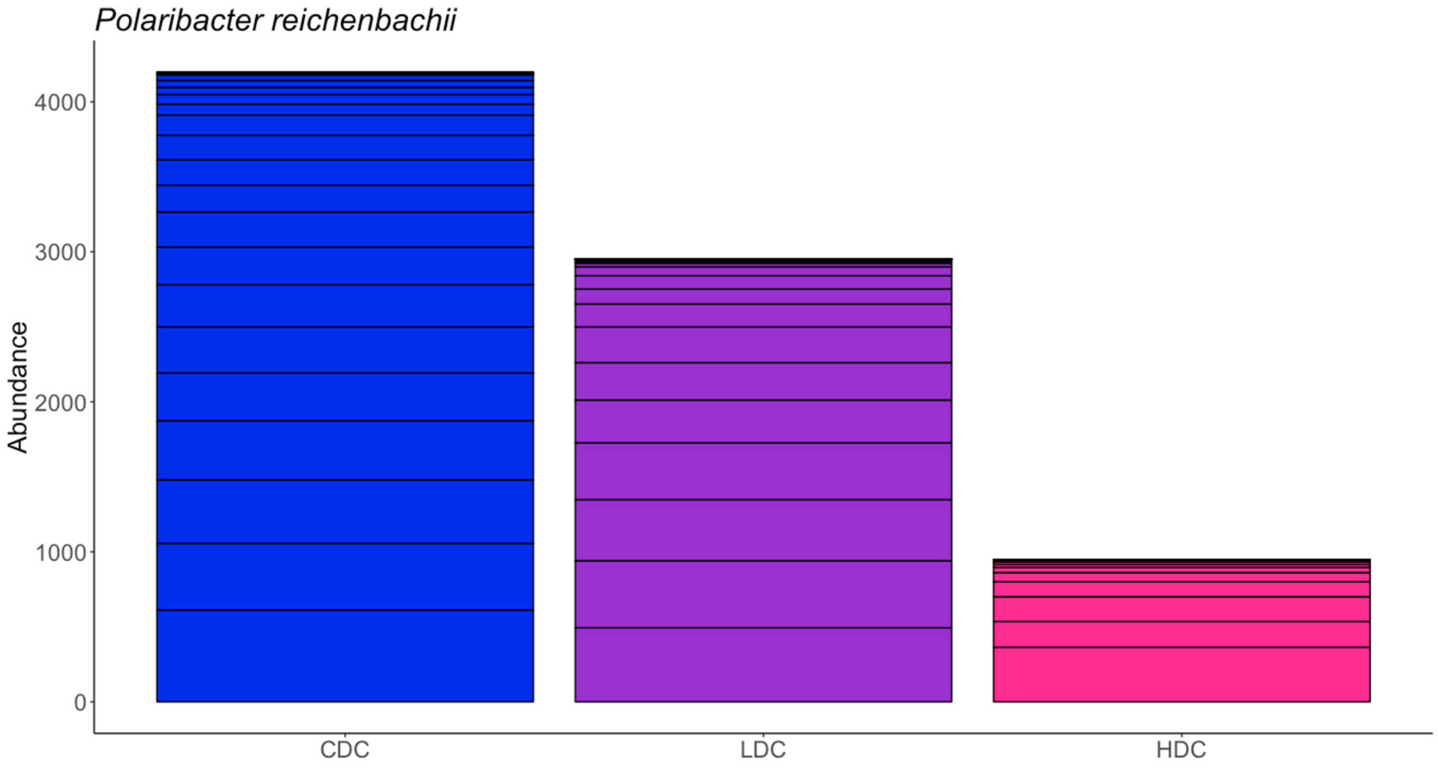

Supplementary Figure 4D

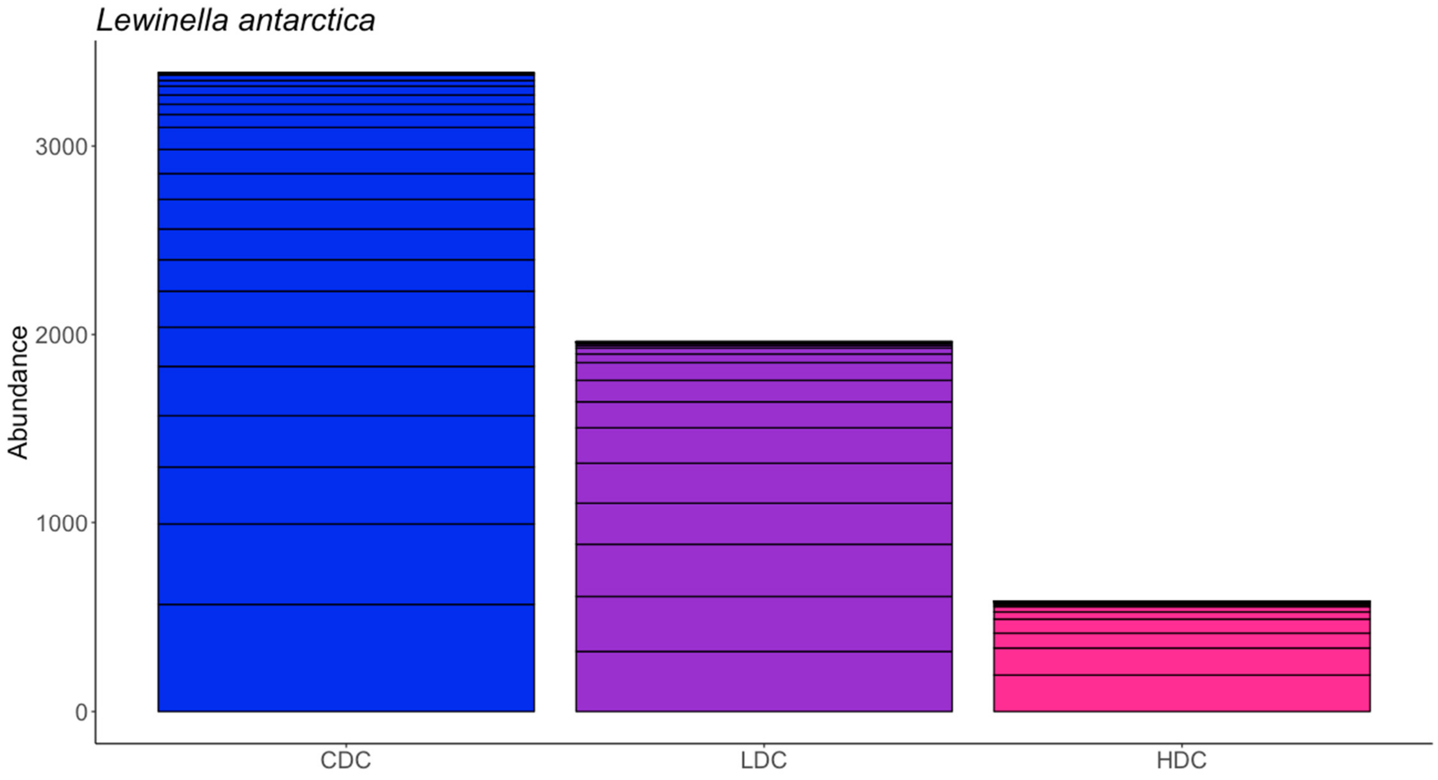

Supplementary Figure 4E

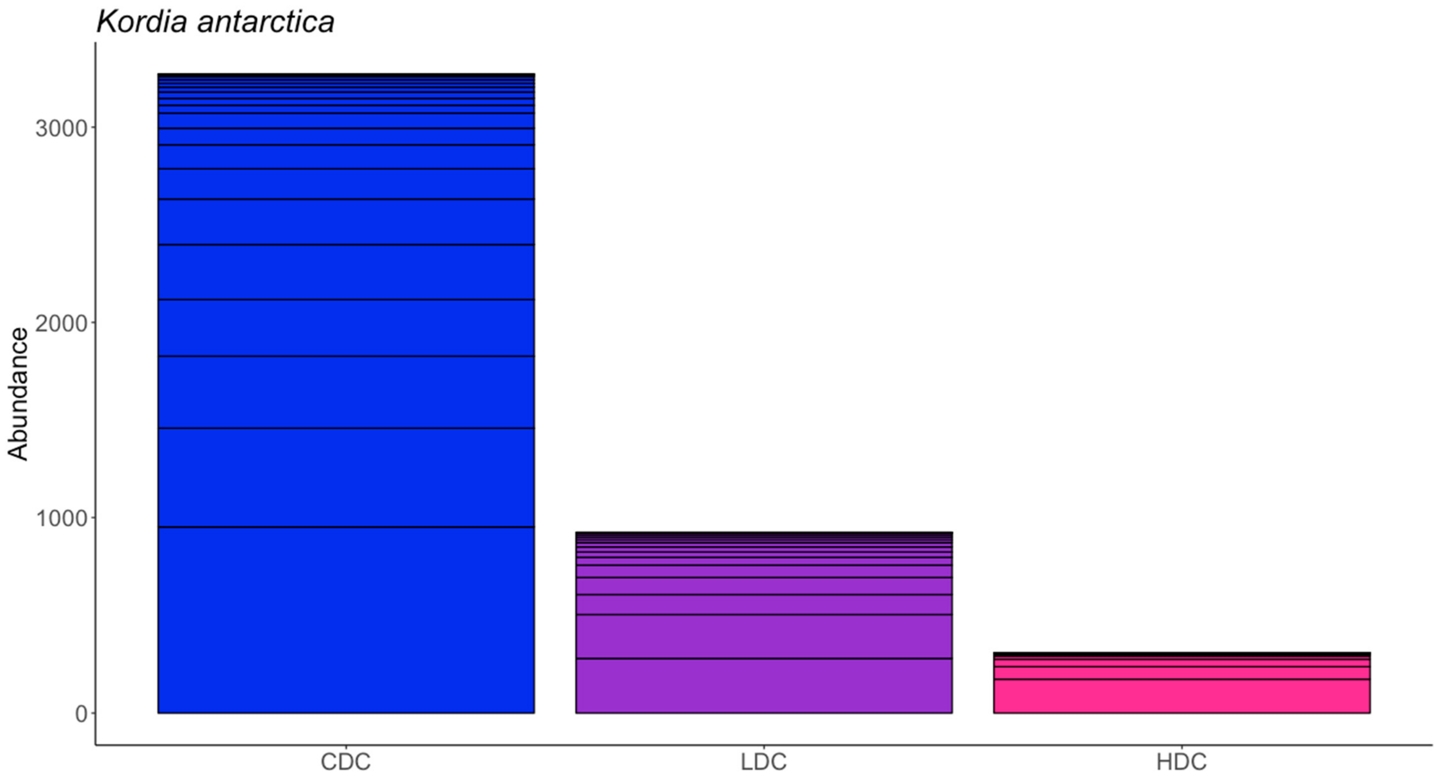

Supplementary Figure 5A

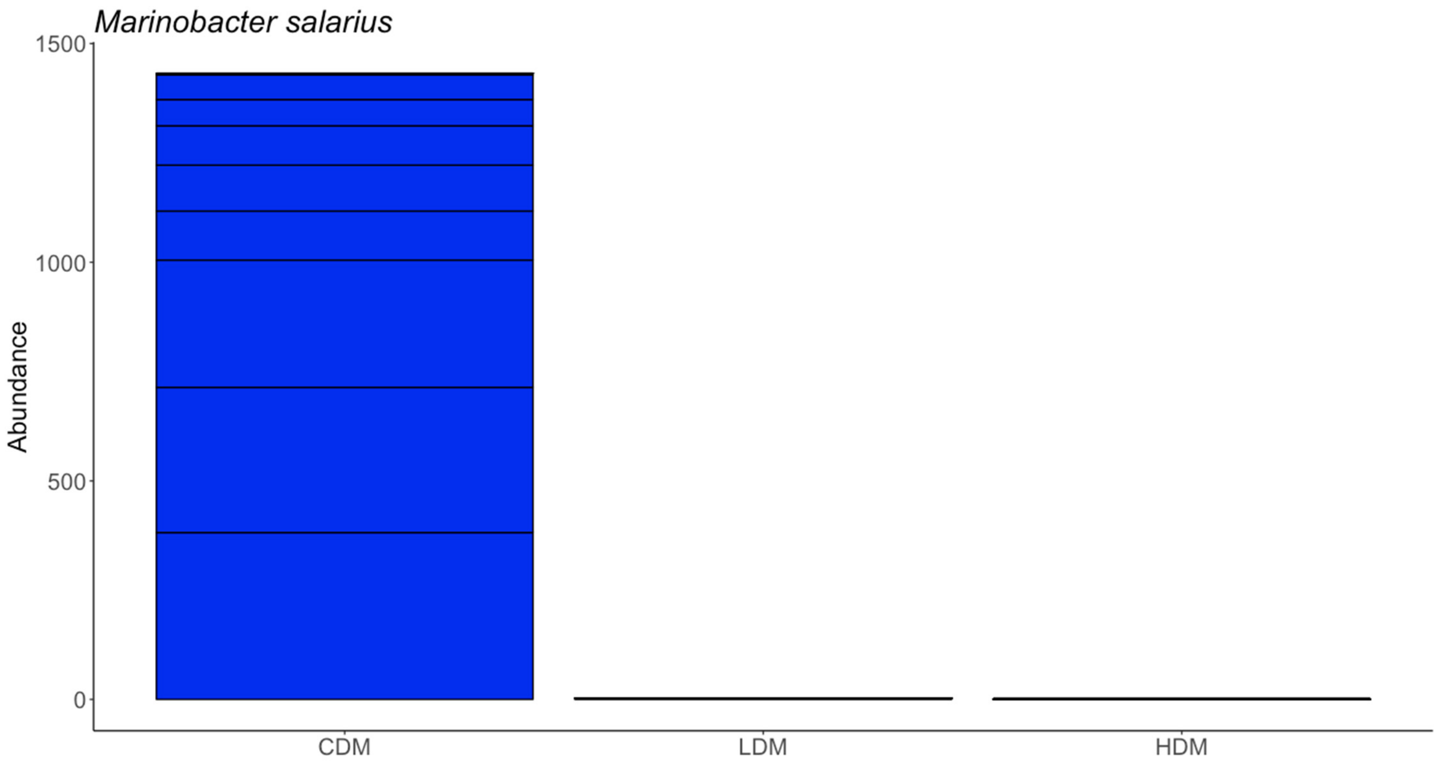

Supplementary Figure 5B

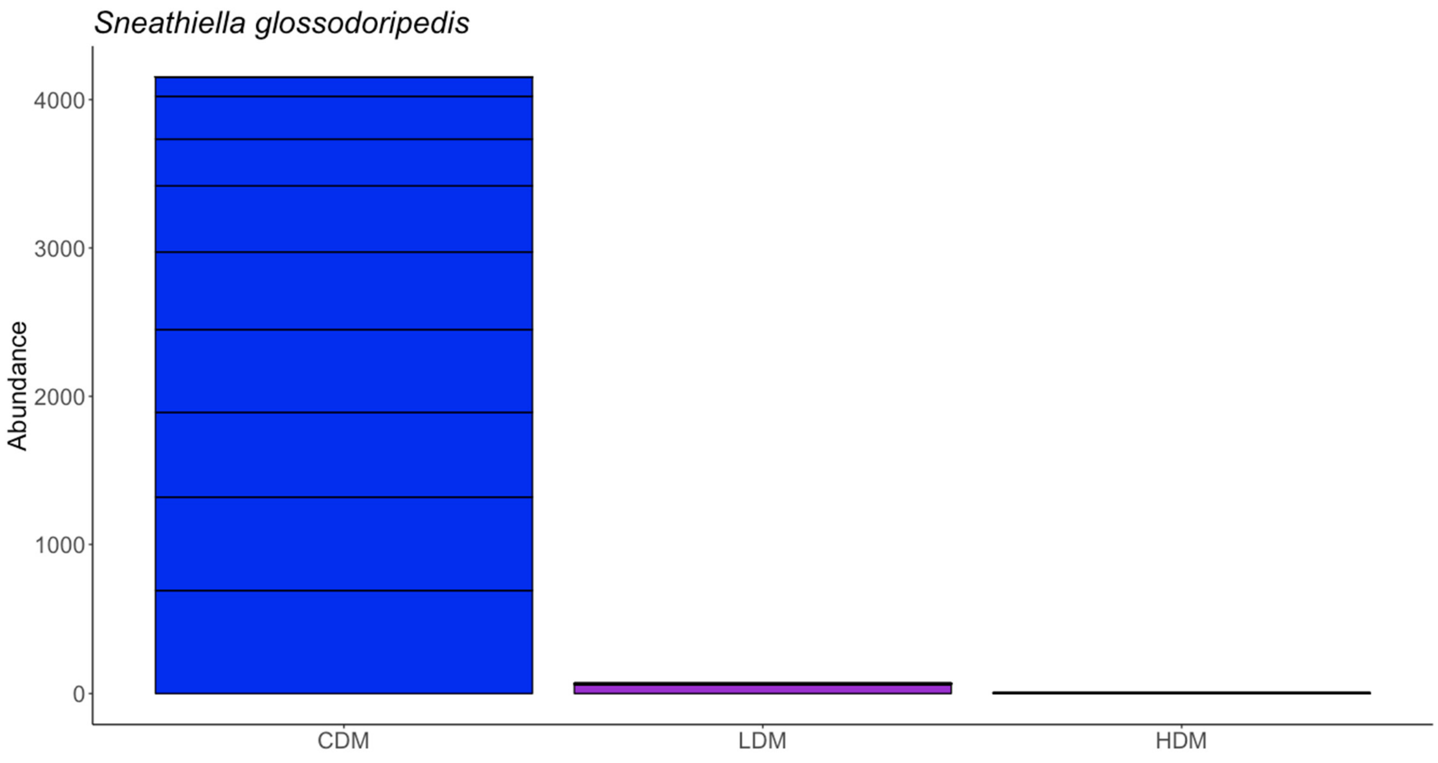

Supplementary Figure 5C

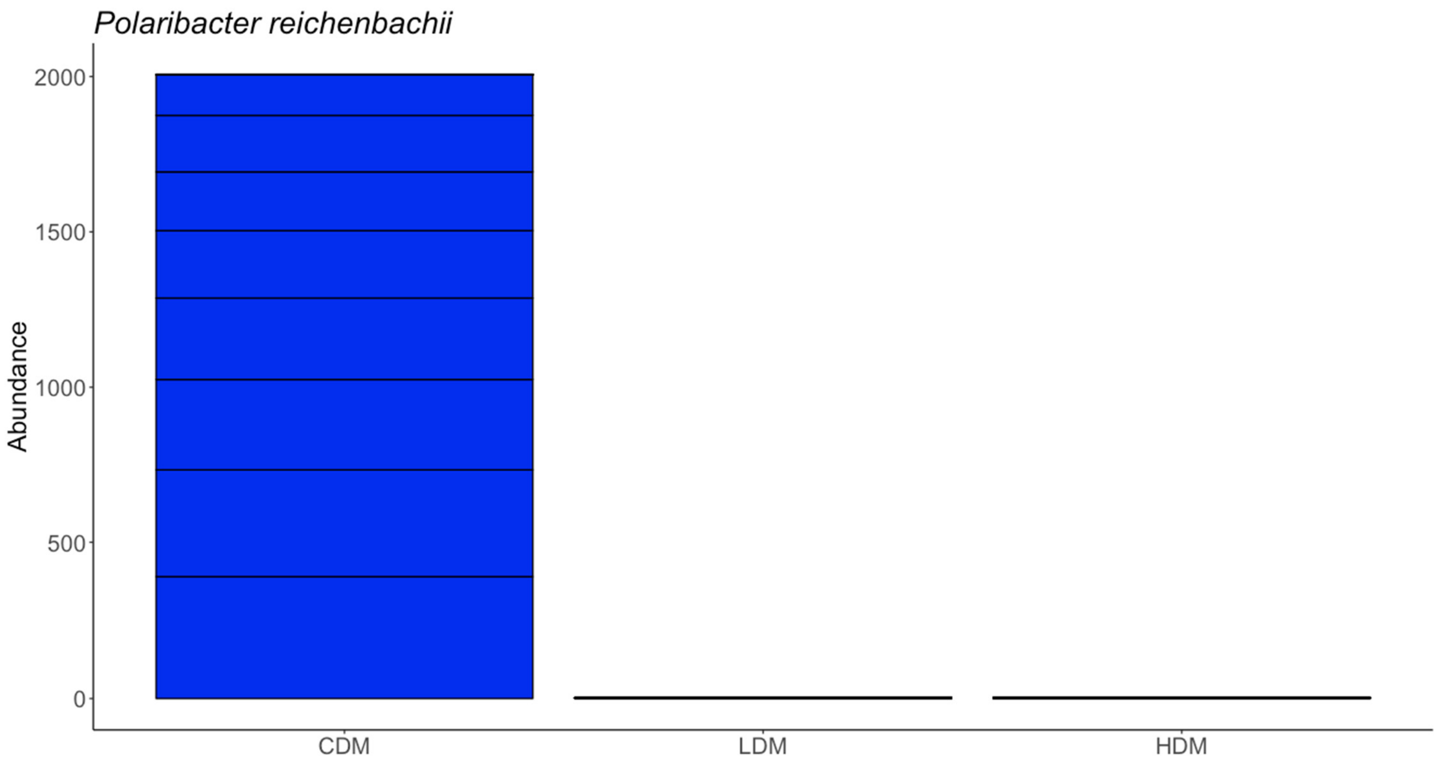

Supplementary Figure 5D

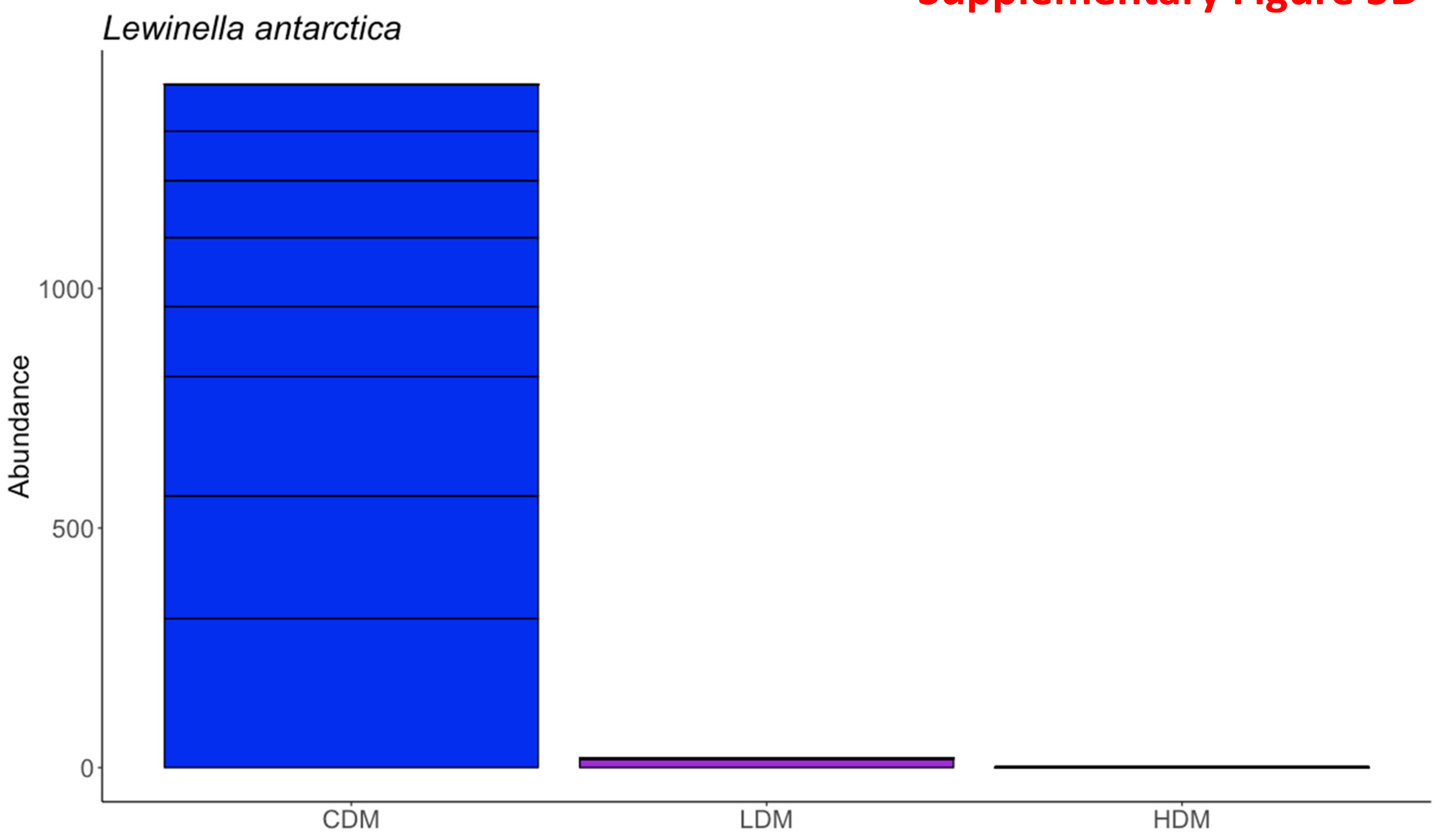

Supplementary Figure 6A

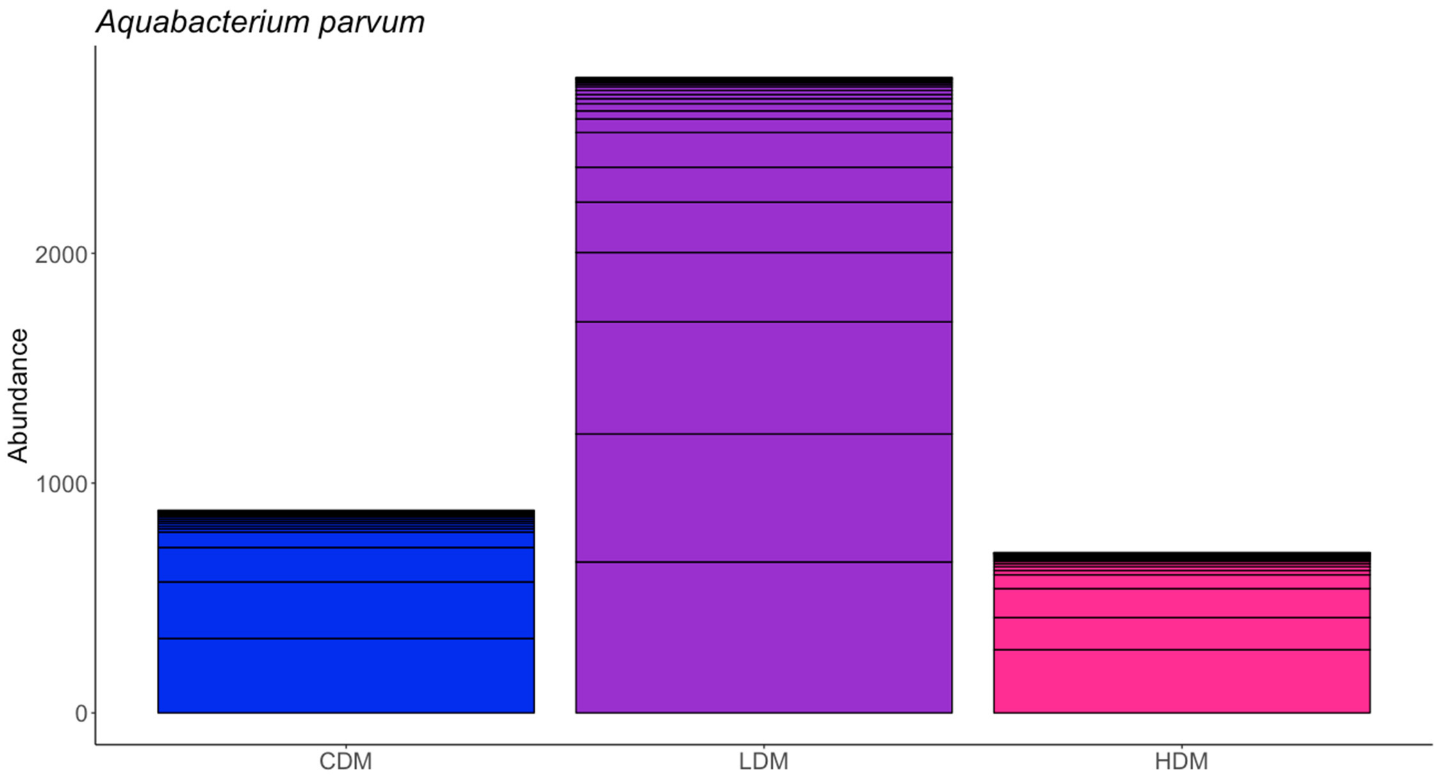

Supplementary Figure 6B

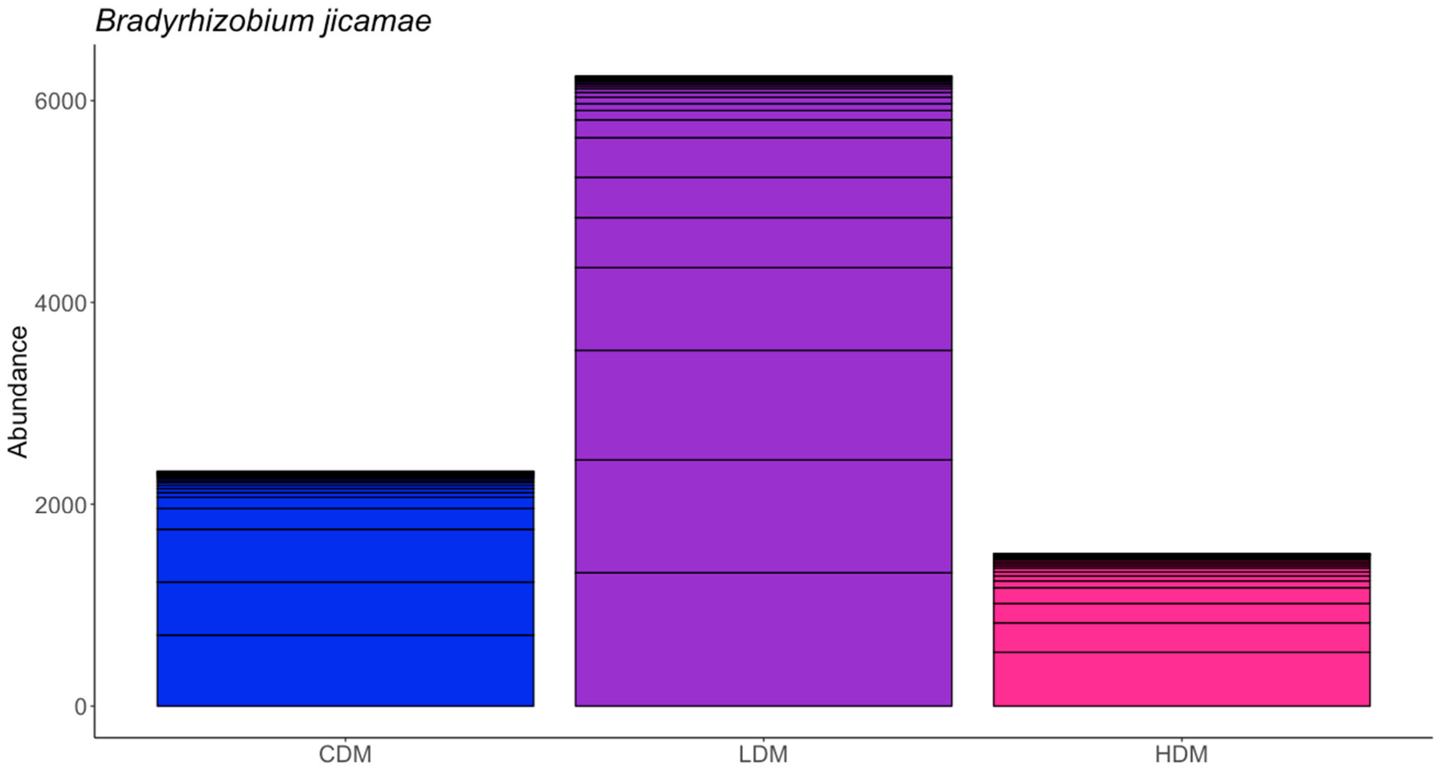

Supplementary Figure 6C

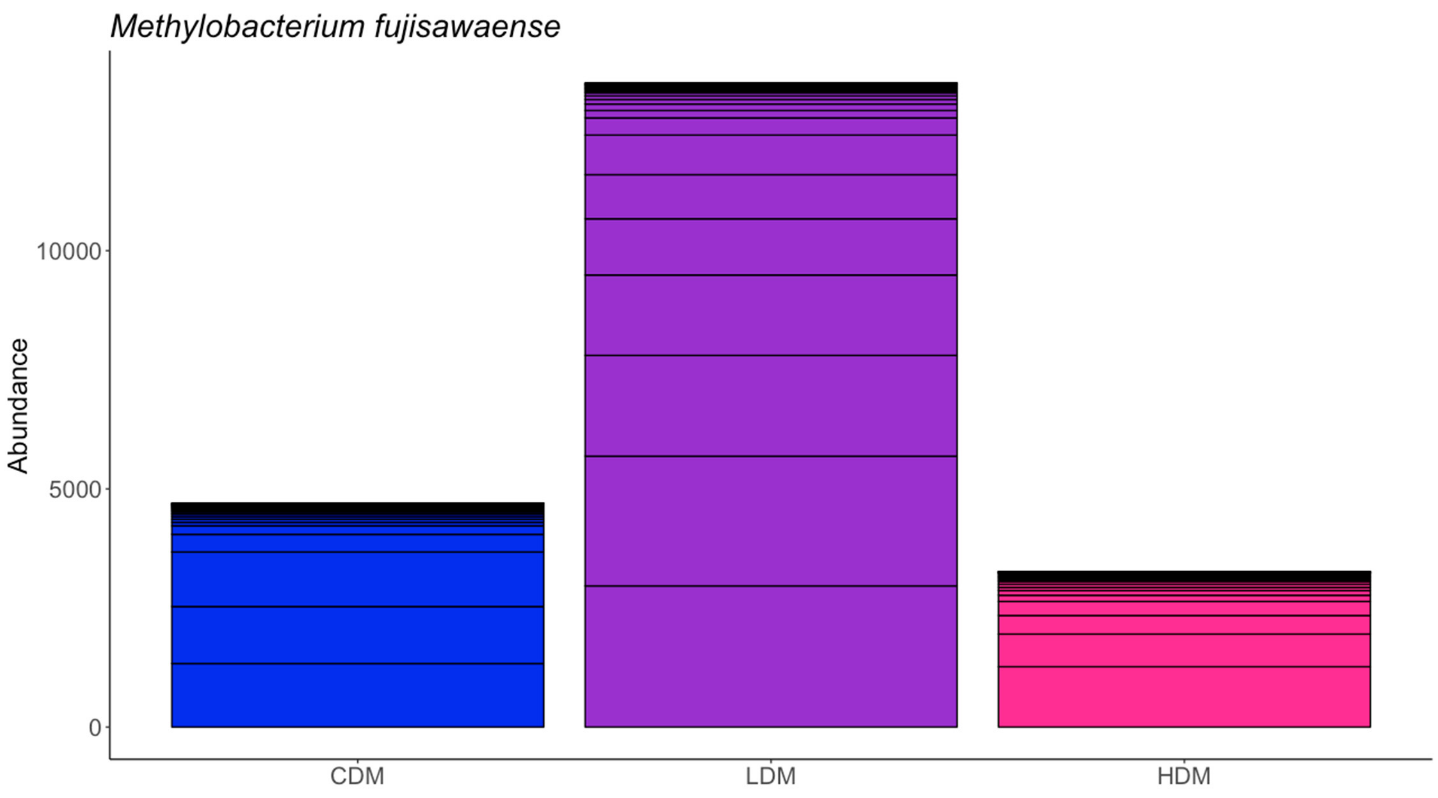

Supplementary Figure 6D

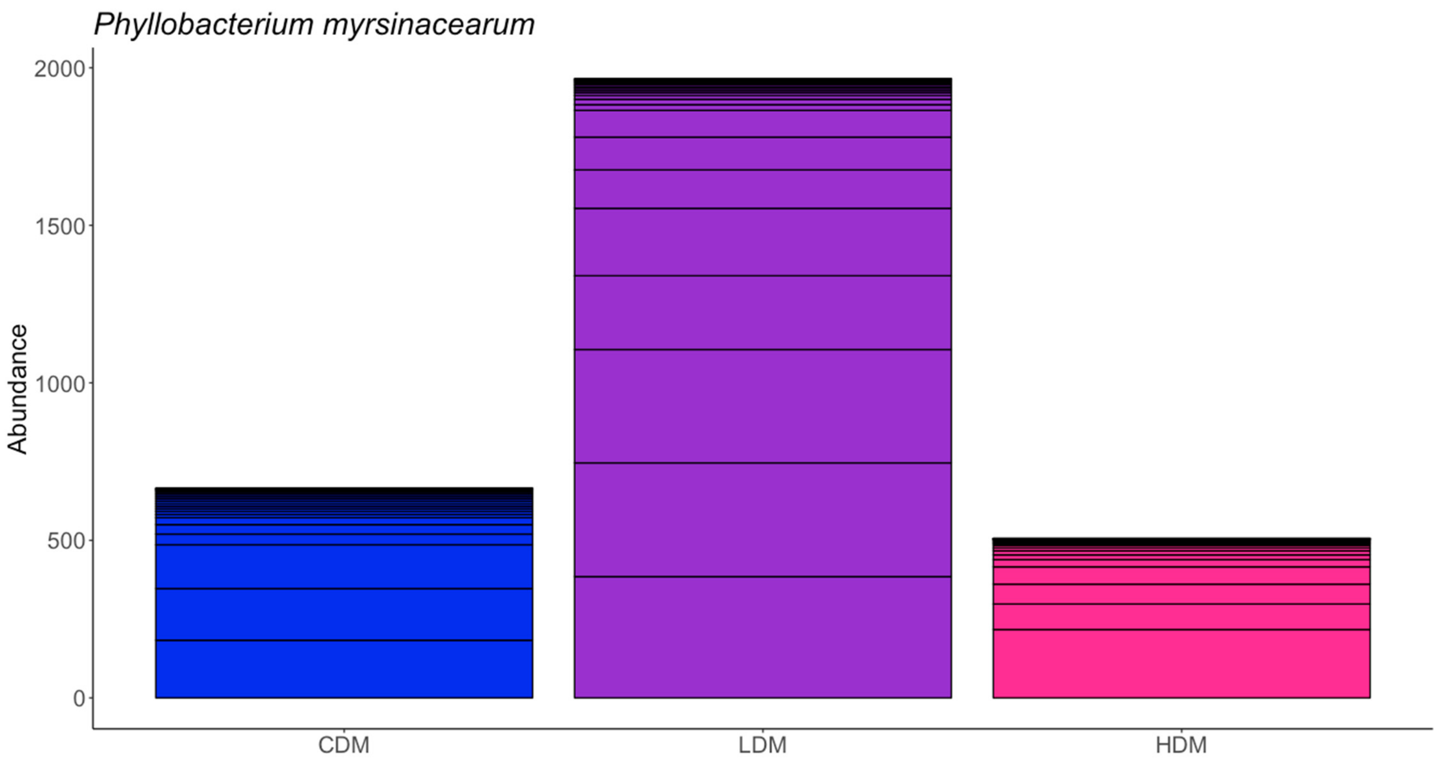

Supplementary Figure 6E

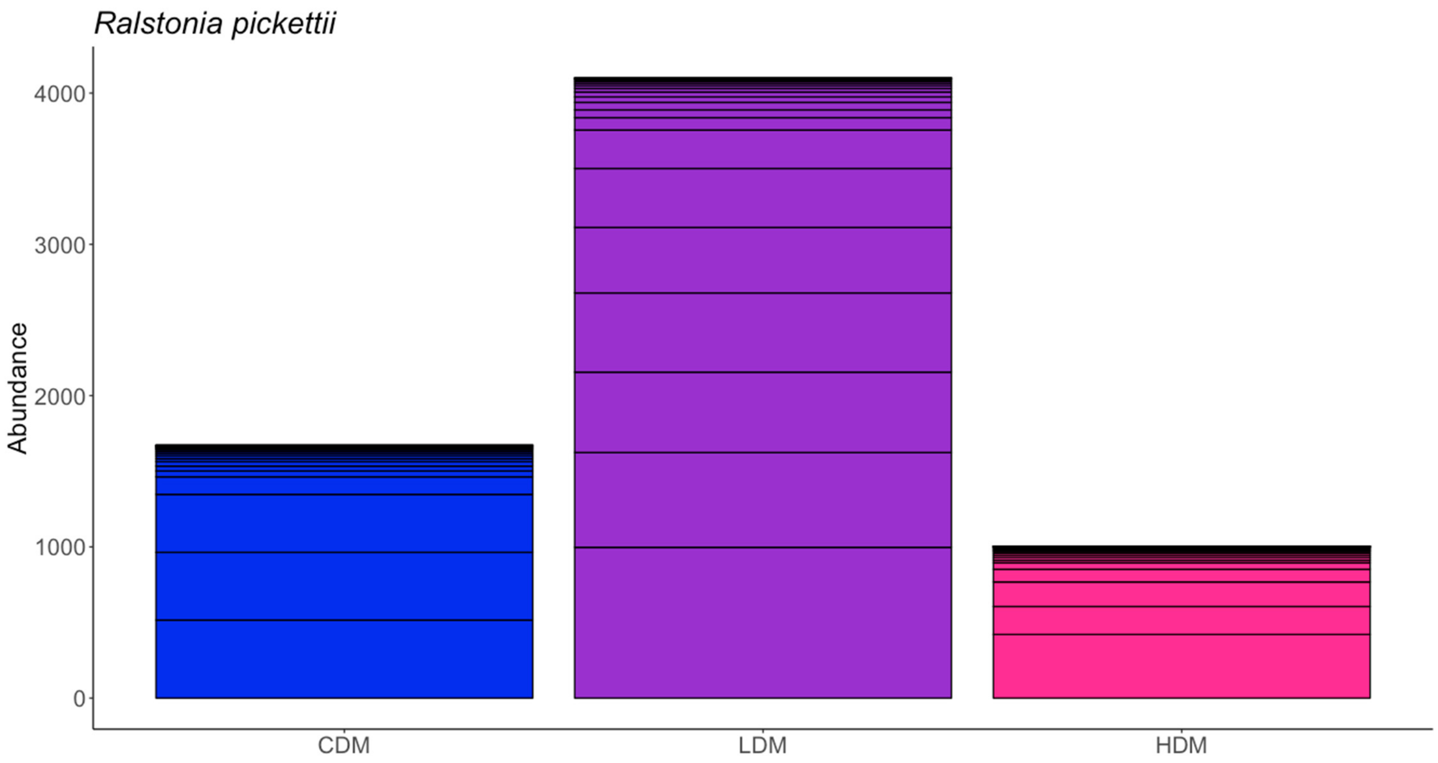

Supplementary Figure 6F

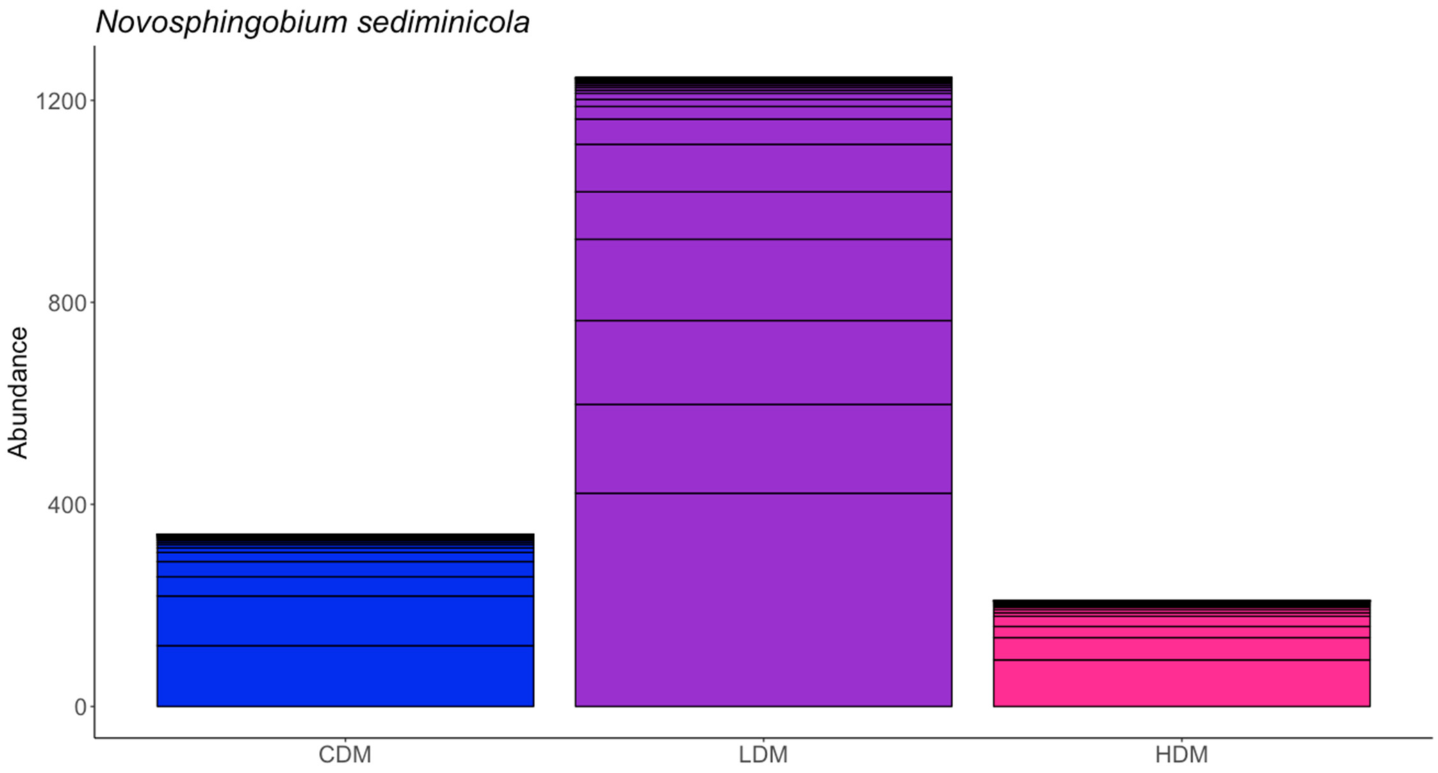

Supplementary Figure 7A

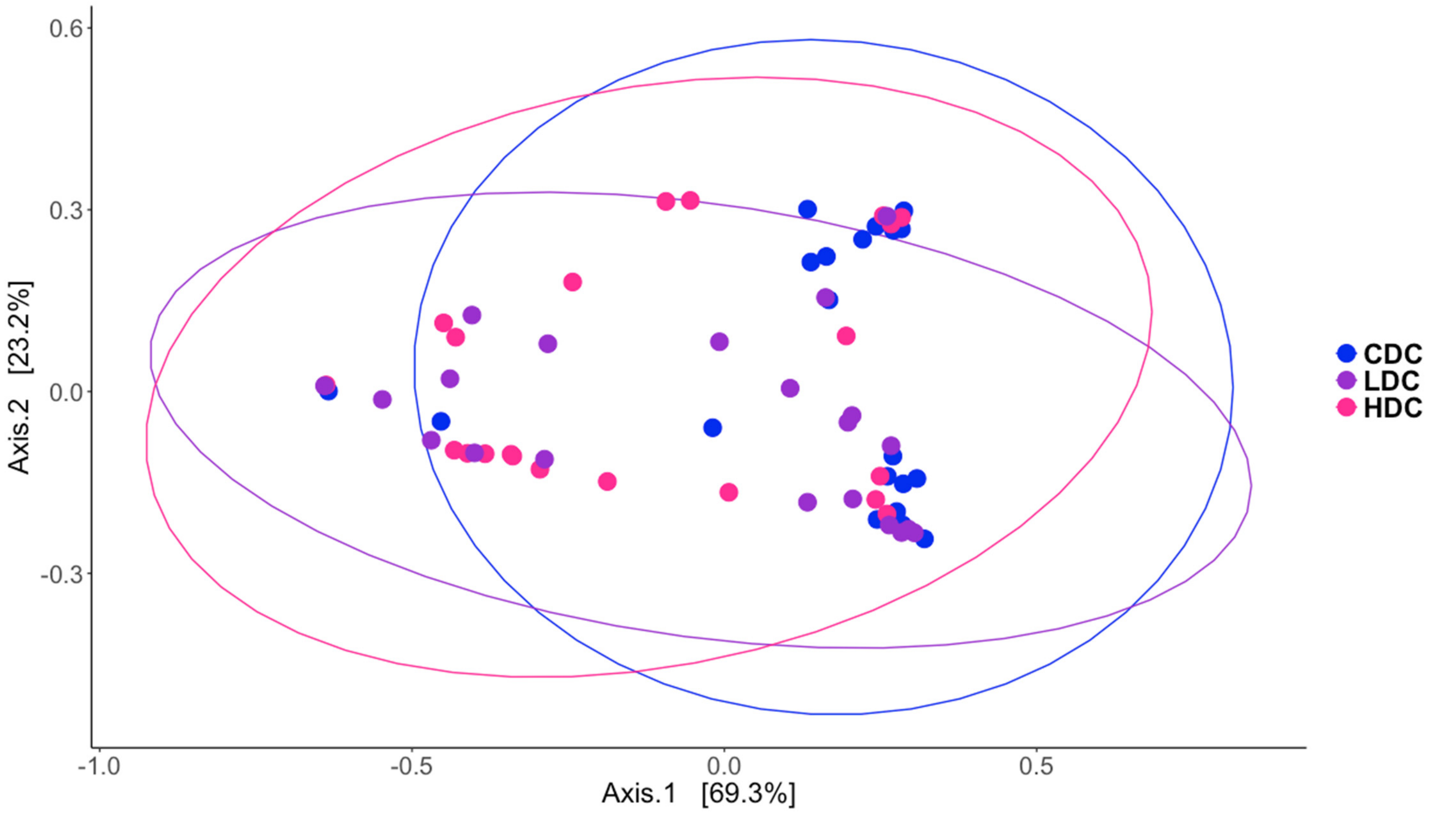

Supplementary Figure 7B

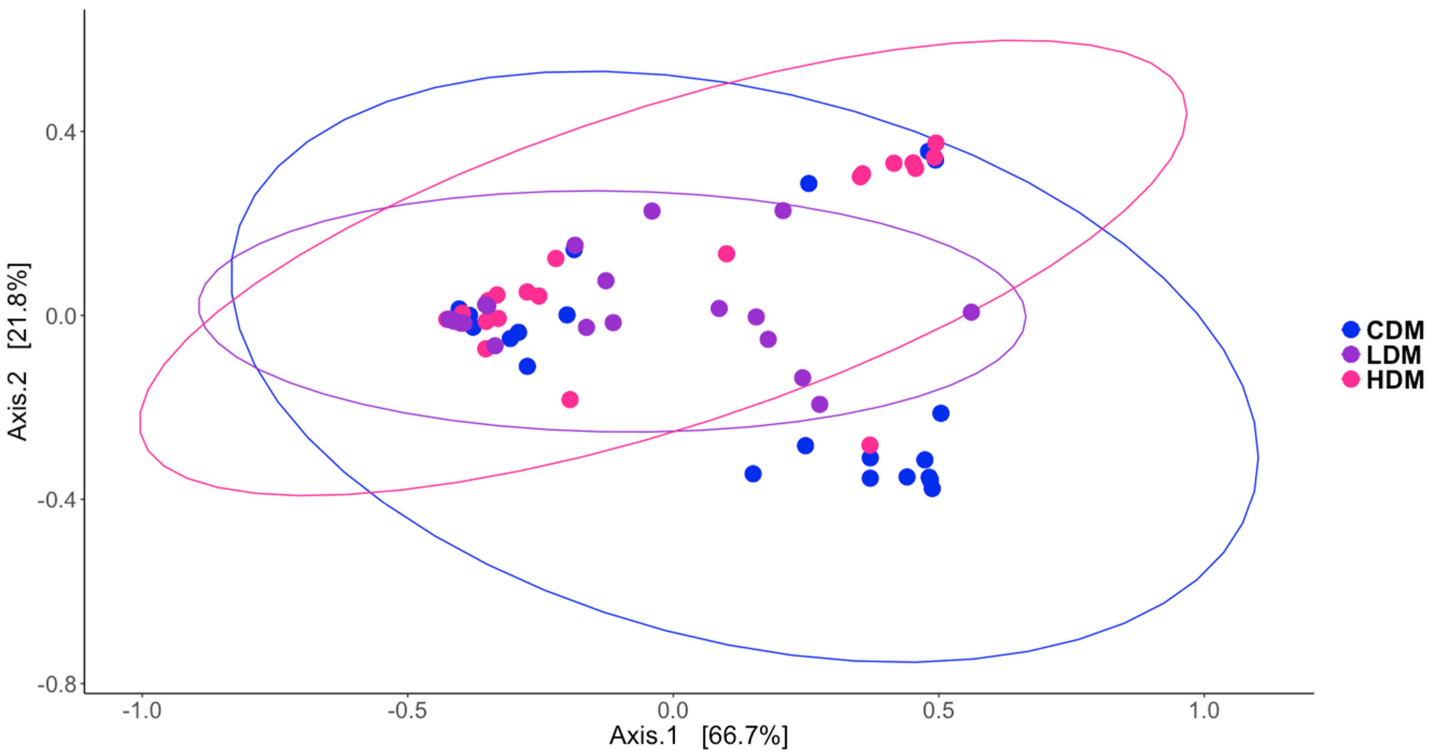

Supplementary Figure 8A

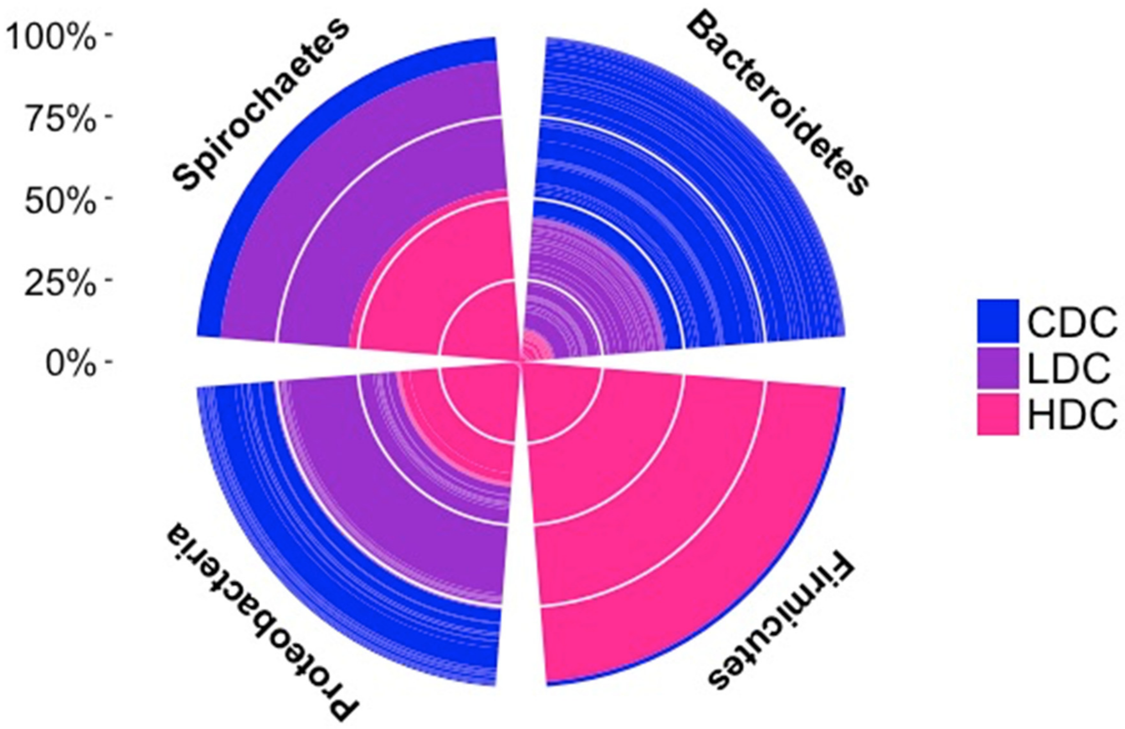

Supplementary Figure 8B

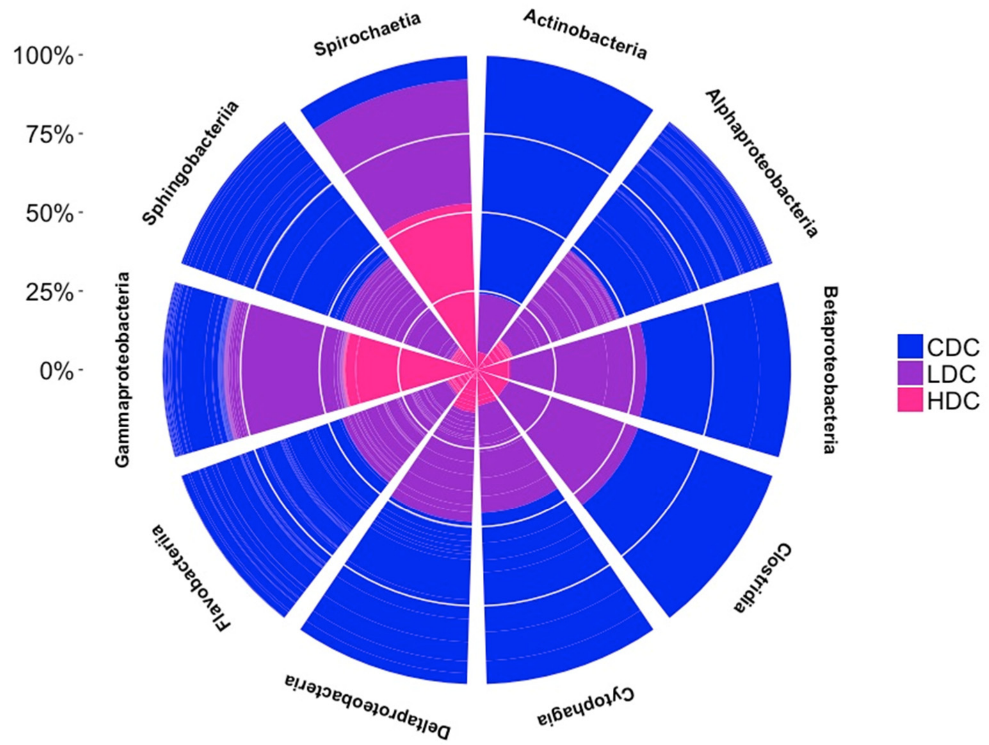

Supplementary Figure 8C

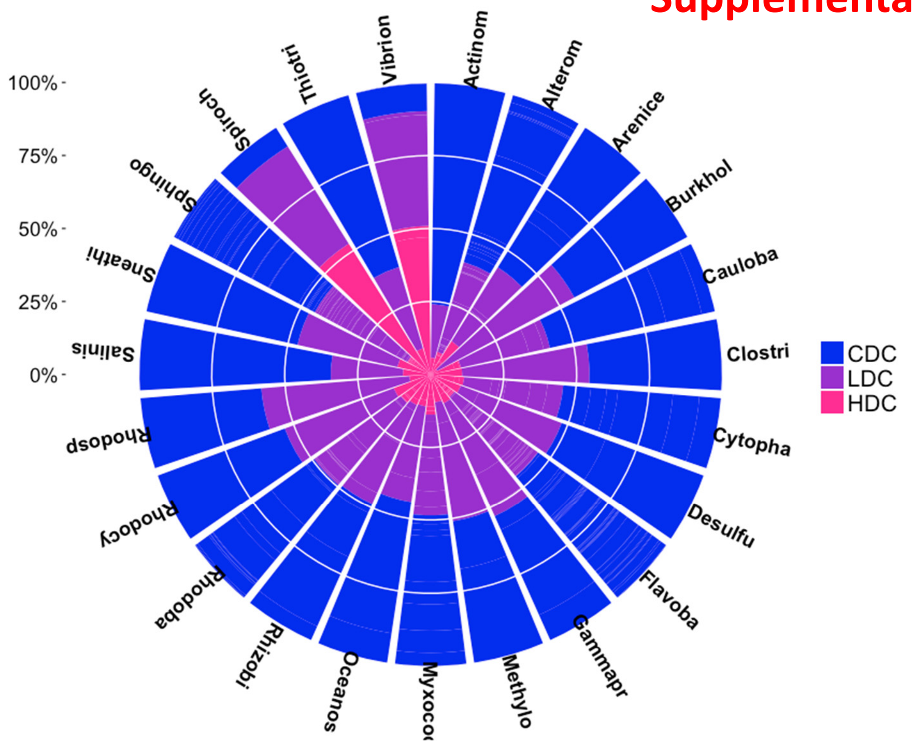

Supplementary Figure 8D

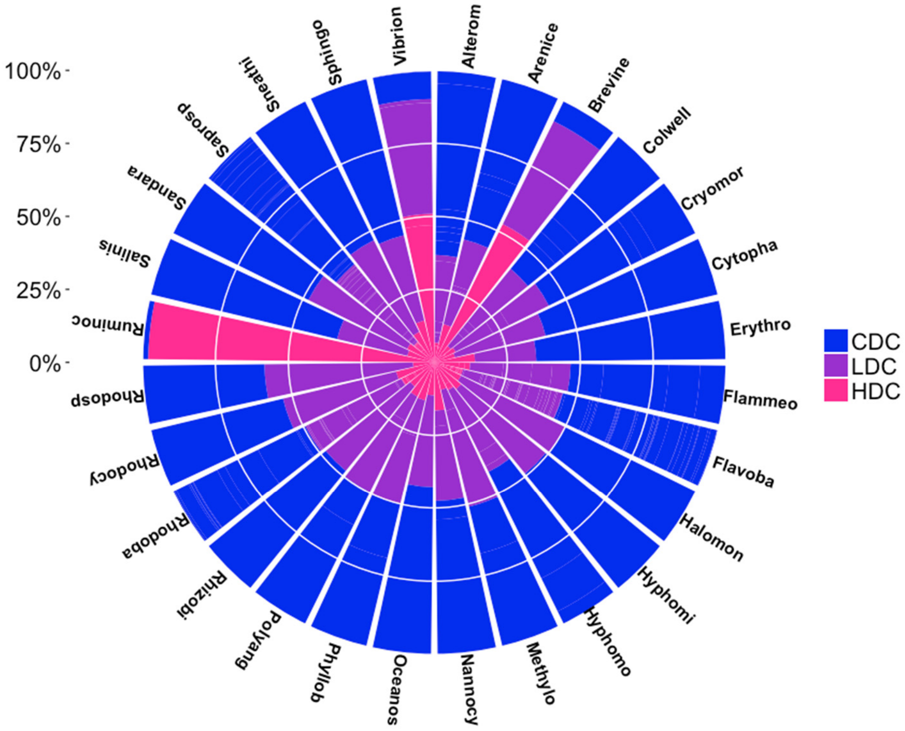

Supplementary Figure 8E

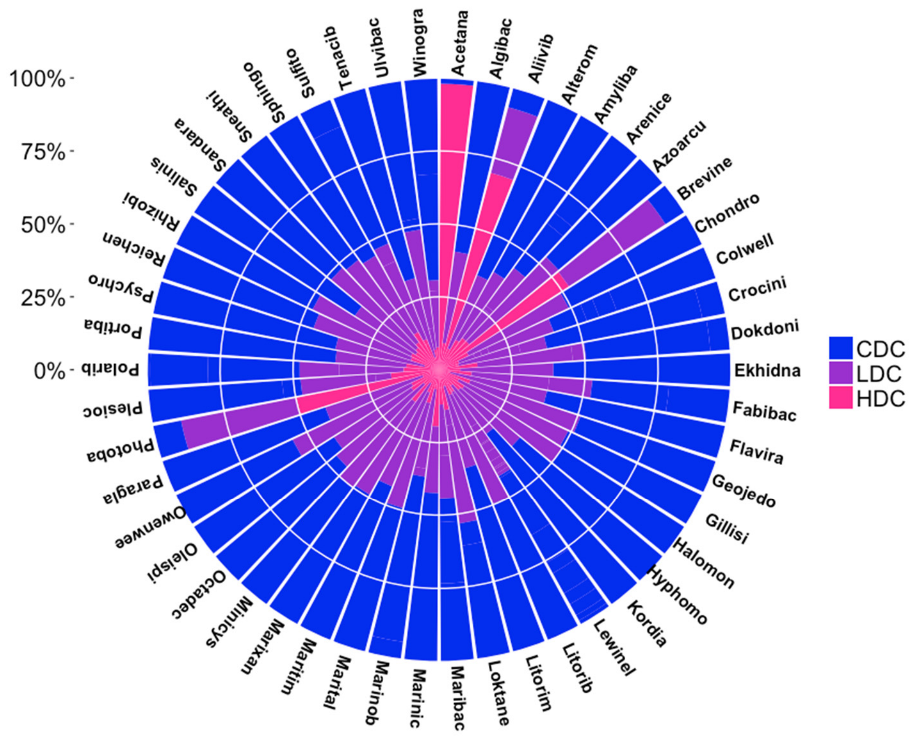

Supplementary Figure 8F

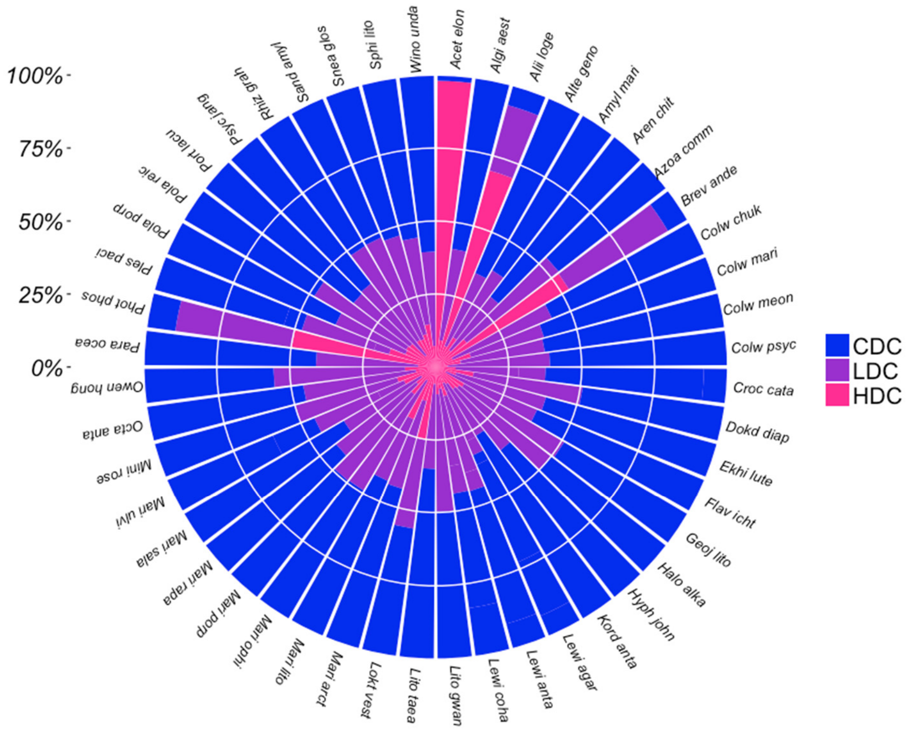

Order

Spiroch	:	Spirochaetales
Sneathi	:	Sneathiellales
Sphingo	:	Sphingobacteriales
Rhodoba	:	Rhodobacterales
Arenice	:	Arenicellales
Alterom	:	Alteromonadales
Flavoba	:	Flavobacteriales
Gammapr:		Gammaproteobacteria_incertae_sedis
Cauloba	:	Caulobacterales
Sphingo	:	Sphingomonadales
Methylo	:	Methylococcales
Cytopha	:	Cytophagales
Myxococ	:	Myxococcales
Salinis	:	Salinisphaerales
Rhizobi	:	Rhizobiales
Vibrion	:	Vibrionales
Desulfu	:	Desulfuromonadales
Clostri	:	Clostridiales
Thiotri	:	Thiotrichales
Rhodocy	:	Rhodocyclales
Oceanos	:	Oceanospirillales
Rhodosp	:	Rhodospirillales
Burkhol	:	Burkholderiales
Actinom	:	Actinomycetales

Family

Brevine	:	Brevinemataceae
Sneathi	:	Sneathiellaceae
Saprosp	:	Saprospiraceae
Rhodoba	:	Rhodobacteraceae
Arenice	:	Arenicellaceae
Alterom	:	Alteromonadaceae
Flavoba	:	Flavobacteriaceae
Hyphomo	:	Hyphomonadaceae
Sphingo	:	Sphingomonadaceae
Methylo	:	Methylococcaceae
Flammeo	:	Flammeovirgaceae
Cryomor	:	Cryomorphaceae
Polyang	:	Polyangiaceae
Nannocy	:	Nannocystaceae
Cytopha	:	Cytophagaceae
Colwell	:	Colwelliaceae
Salinis	:	Salinisphaeraceae
Phyllob	:	Phyllobacteriaceae
Erythro	:	Erythrobacteraceae
Vibrion	:	Vibrionaceae
Ruminoc	:	Ruminococcaceae
Rhodocy	:	Rhodocyclaceae
Sandara	:	Sandaracinaceae
Oceanos	:	Oceanospirillaceae
Rhodosp	:	Rhodospirillaceae
Halomon	:	Halomonadaceae
Hyphomi	:	Hyphomicrobiaceae
Rhizobi	:	Rhizobiaceae

Genus

Brevine	:	*Brevinema*
Sneathi	:	*Sneathiella*
Arenice	:	*Arenicella*
Alterom	:	*Alteromonas*
Maribac	:	*Maribacter*
Sphingo	:	*Sphingorhabdus*
Polarib	:	*Polaribacter*
Flavira	:	*Flaviramulus*
Litorib	:	*Litoribaculum*
Fabibac	:	*Fabibacter*
Winogra	:	*Winogradskyella*
Kordia	:	*Kordia*
Crocini	:	*Crocinitomix*
Lewinel	:	*Lewinella*
Sulfito	:	*Sulfitobacter*
Chondro	:	*Chondromyces*
Ulvibac	:	*Ulvibacter*
Plesioc	:	*Plesiocystis*
Gillisi	:	*Gillisia*
Ekhidna	:	*Ekhidna*
Colwell	:	*Colwellia*
Hyphomo	:	*Hyphomonas*
Marinic	:	*Marinicella*
Salinis	:	*Salinisphaera*
Litorim	:	*Litorimonas*
Paragla	:	*Paraglaciecola*
Marinob	:	*Marinobacter*
Algibac	:	*Algibacter*
Psychro	:	*Psychroserpens*
Portiba	:	*Portibacter*
Photoba	:	*Photobacterium*
Dokdoni	:	*Dokdonia*
Acetana	:	*Acetanaerobacterium*
Aliivib	:	*Aliivibrio*
Marixan	:	*Marixanthomonas*
Loktane	:	*Loktanella*
Octadec	:	*Octadecabacter*
Amyliba	:	*Amylibacter*
Minicys	:	*Minicystis*
Azoarcu	:	*Azoarcus*
Owenwee	:	*Owenweeksia*
Maritim	:	*Maritimimonas*
Sandara	:	*Sandaracinus*
Oleispi	:	*Oleispira*
Halomon	:	*Halomonas*
Marital	:	*Maritalea*
Geojedo	:	*Geojedonia*
Rhizobi	:	*Rhizobium*
Reichen	:	*Reichenbachiella*
Tenacib	:	*Tenacibaculum*

Species

Brev ande	:	*Brevinema andersonii*
Snea glos	:	*Sneathiella glossodoripedis*
Alte geno	:	*Alteromonas genovensis*
Mari ulvi	:	*Maribacter ulvicola*
Sphi lito	:	*Sphingorhabdus litoris*
Pola reic	:	*Polaribacter reichenbachii*
Flav icht	:	*Flaviramulus ichthyoenteri*
Lito gwan	:	*Litoribaculum gwangyangense*
Wino unda	:	*Winogradskyella undariae*
Kord anta	:	*Kordia antarctica*
Croc cata	:	*Crocinitomix catalasitica*
Lewi agar	:	*Lewinella agarilytica*
Ples paci	:	*Plesiocystis pacifica*
Ekhi lute	:	*Ekhidna lutea*
Colw meon	:	*Colwellia meonggei*
Colw psyc	:	*Colwellia psychrerythraea*
Hyph john	:	*Hyphomonas johnsonii*
Lewi anta	:	*Lewinella antarctica*
Mari lito	:	*Marinicella litoralis*
Lito taea	:	*Litorimonas taeanensis*
Para ocea	:	*Paraglaciecola oceanifecundans*
Mari sala	:	*Marinobacter salarius*
Algi aest	:	*Algibacter aestuarii*
Psyc jang	:	*Psychroserpens jangbogonensis*
Lewi coha	:	*Lewinella cohaerens*
Port lacu	:	*Portibacter lacus*
Phot phos	:	*Photobacterium phosphoreum*
Dokd diap	:	*Dokdonia diaphoros*
Acet elon	:	*Acetanaerobacterium elongatum*
Alii loge	:	*Aliivibrio logei*
Mari ophi	:	*Marixanthomonas ophiurae*
Lokt vest	:	*Loktanella vestfoldensis*
Octa anta	:	*Octadecabacter antarcticus*
Colw mari	:	*Colwellia maris*
Amyl mari	:	*Amylibacter marinus*
Mini rose	:	*Minicystis rosea*
Azoa comm	:	*Azoarcus communis*
Owen hong	:	*Owenweeksia hongkongensis*
Colw chuk	:	*Colwellia chukchiensis*
Mari rapa	:	*Maritimimonas rapanae*
Aren chit	:	*Arenicella chitinivorans*
Pola porp	:	*Polaribacter porphyrae*
Sand amyl	:	*Sandaracinus amylolyticus*
Mari arct	:	*Maribacter arcticus*
Halo alka	:	*Halomonas alkaliphila*
Mari porp	:	*Maritalea porphyrae*
Geoj lito	:	*Geojedonia litorea*
Rhiz grah	:	*Rhizobium grahamii*

Supplementary Figure 8 abbreviations

Supplementary Figure 9A

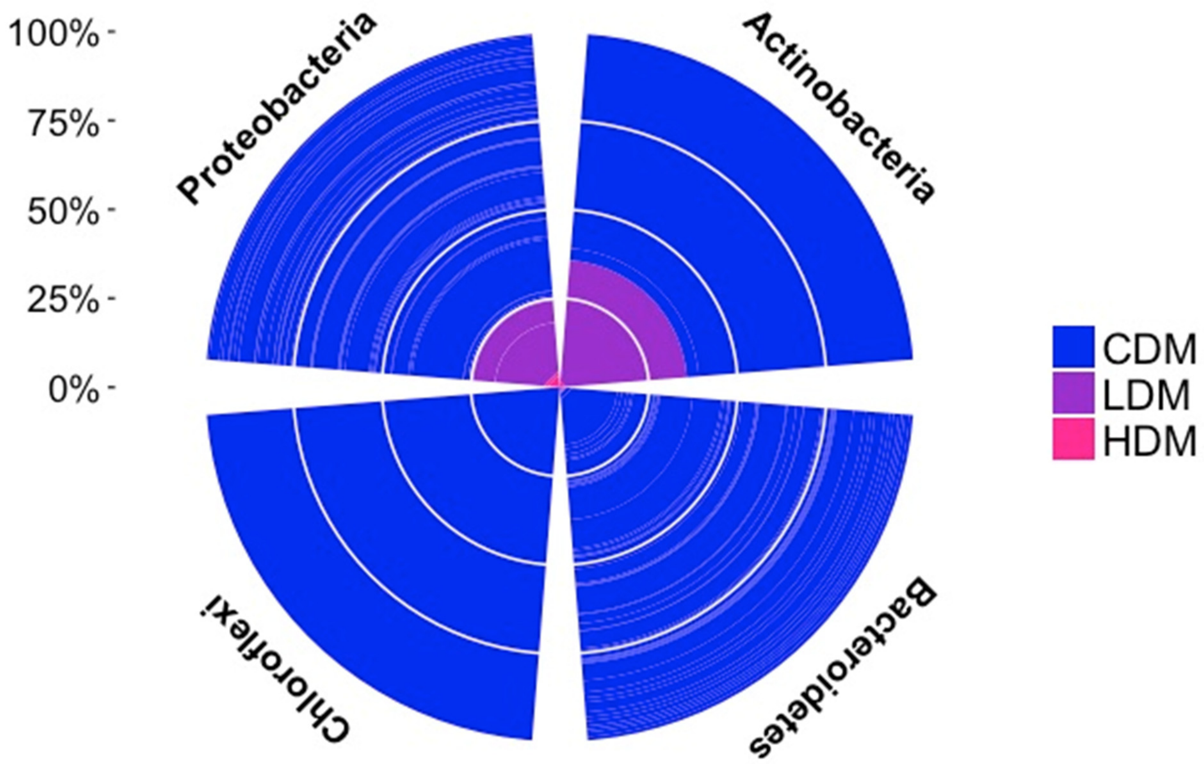

Supplementary Figure 9B

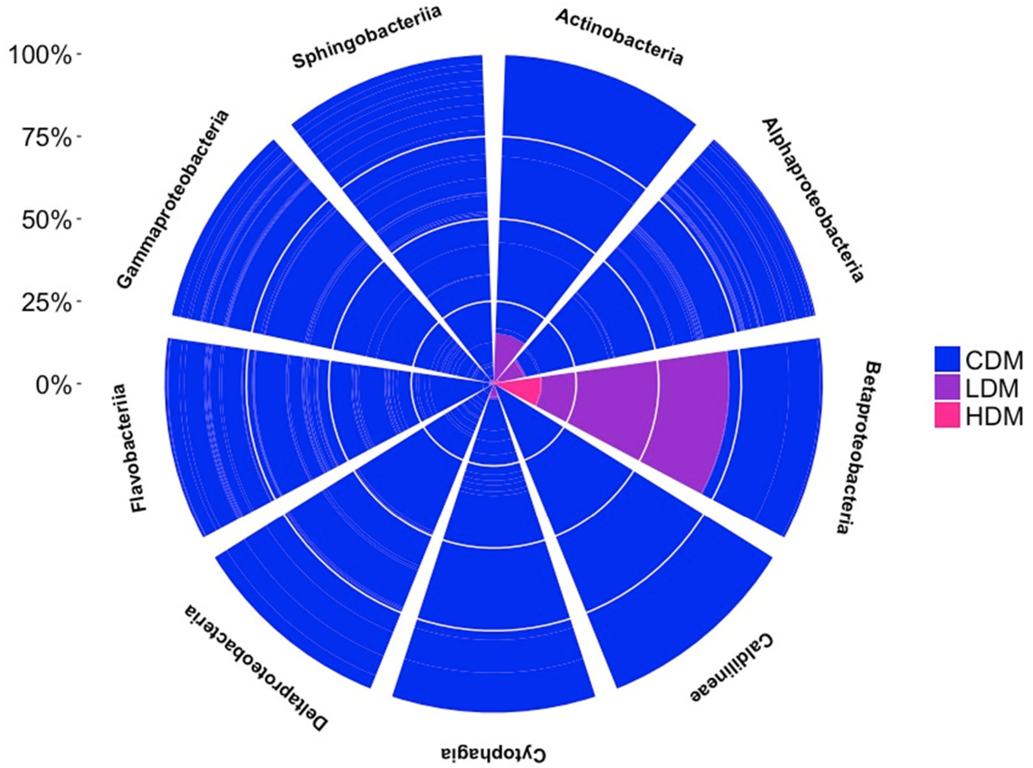

Supplementary Figure 9C

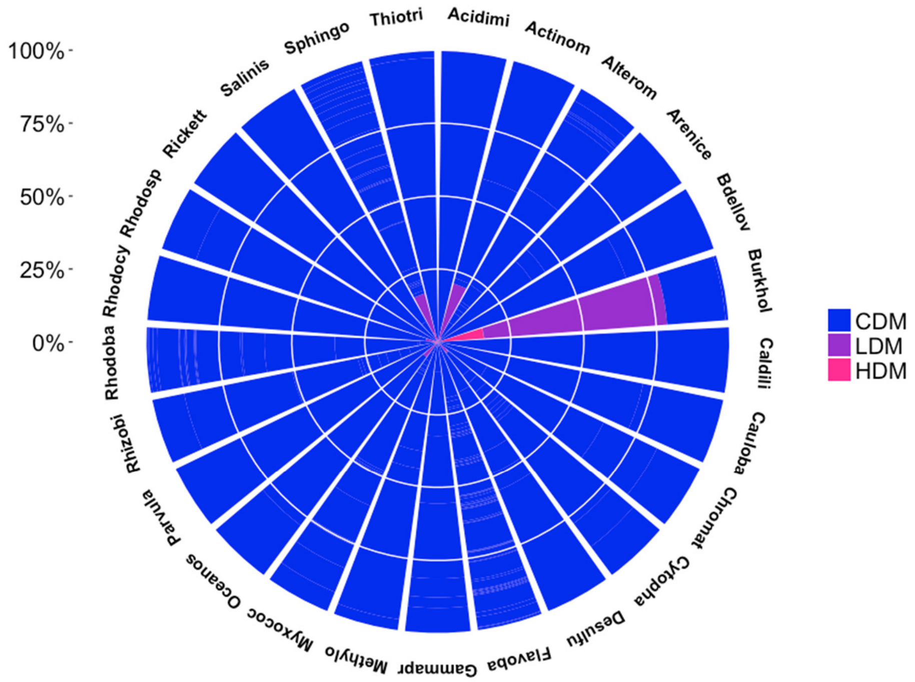

Supplementary Figure 9D

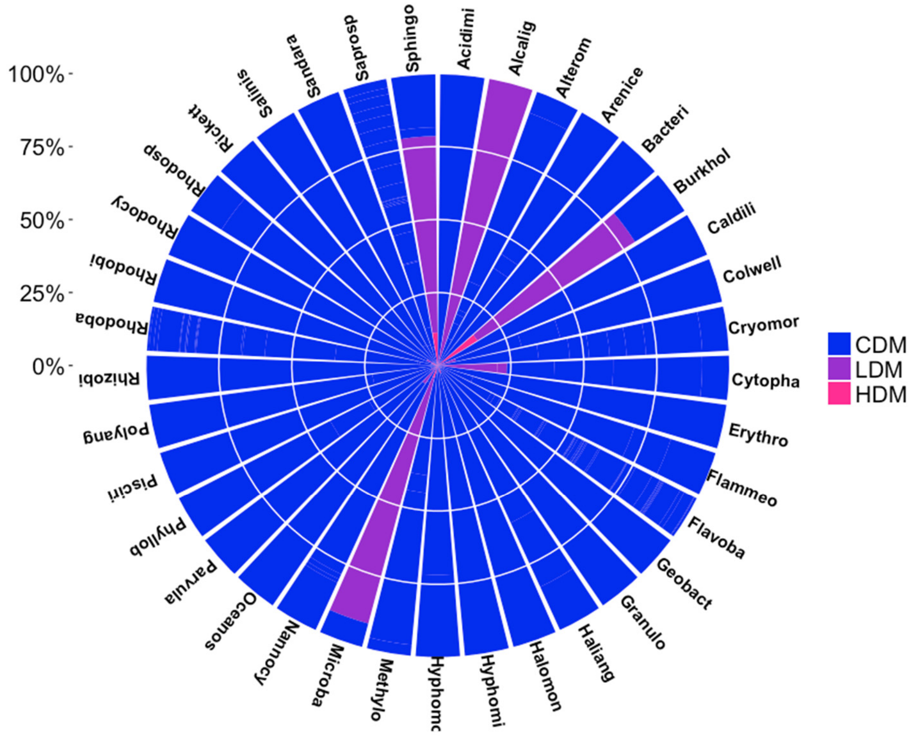

Supplementary Figure 9E

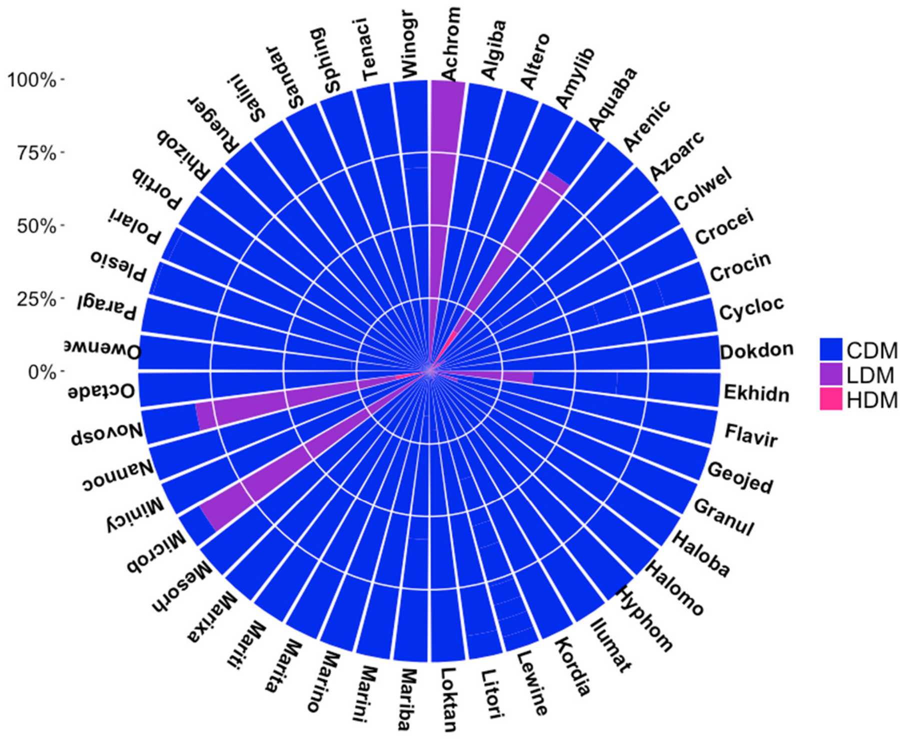

Supplementary Figure 9F

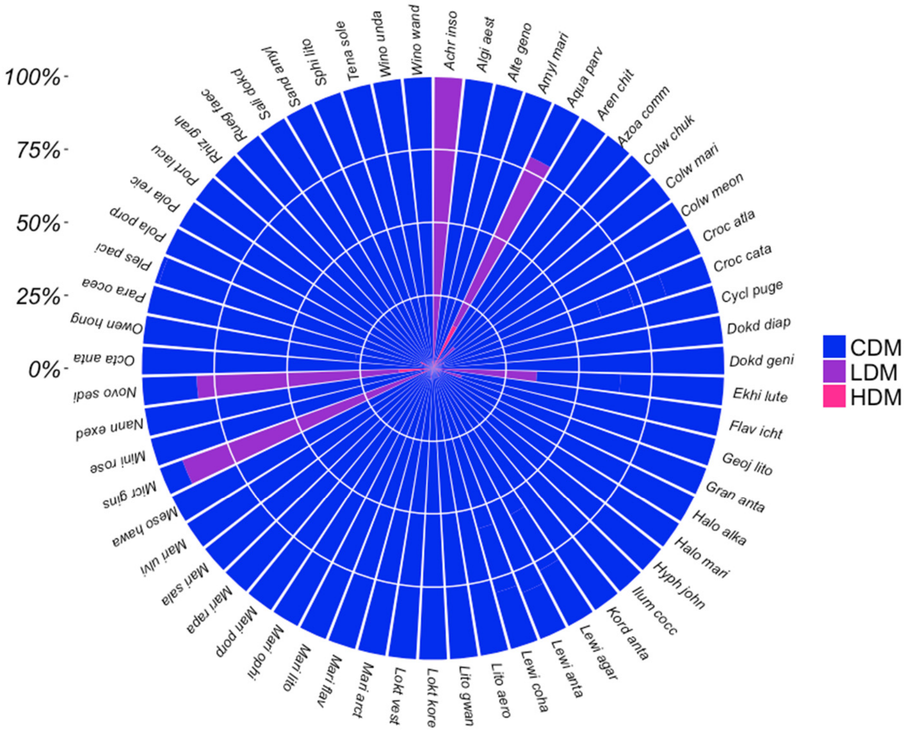

Supplementary Figure 9 abbreviations

Order

Actinom	:	Actinomycetales
Alterom	:	Alteromonadales
Flavoba	:	Flavobacteriales
Sphingo	:	Sphingomonadales
Sphingo	:	Sphingobacteriales
Myxococ	:	Myxococcales
Cytopha	:	Cytophagales
Burkhol	:	Burkholderiales
Cauloba	:	Caulobacterales
Gammapr	:	Gammaproteobacteria_incertae_sedis
Salinis	:	Salinisphaerales
Acidimi	:	Acidimicrobiales
Rhodoba	:	Rhodobacterales
Rhizobi	:	Rhizobiales
Chromat	:	Chromatiales
Caldili	:	Caldilineales
Thiotri	:	Thiotrichales
Bdellov	:	Bdellovibrionales
Rhodocy	:	Rhodocyclales
Arenice	:	Arenicellales
Oceanos	:	Oceanospirillales

Family

Saprosp	:	Saprospiraceae
Microba	:	Microbacteriaceae
Rhodoba	:	Rhodobacteraceae
Arenice	:	Arenicellaceae
Alterom	:	Alteromonadaceae
Flavoba	:	Flavobacteriaceae
Hyphomo	:	Hyphomonadaceae
Sphingo	:	Sphingomonadaceae
Methylo	:	Methylococcaceae
Flammeo	:	Flammeovirgaceae
Cryomor	:	Cryomorphaceae
Polyang	:	Polyangiaceae
Nannocy	:	Nannocystaceae
Parvula	:	Parvularculaceae
Cytopha	:	Cytophagaceae
Rhodobi	:	Rhodobiaceae
Colwell	:	Colwelliaceae
Alcalig	:	Alcaligenaceae
Burkhol	:	Burkholderiales_incertae_sedis
Salinis	:	Salinisphaeraceae
Acidimi	:	Acidimicrobiaceae
Phyllob	:	Phyllobacteriaceae
Rickett	:	Rickettsiaceae
Haliang	:	Haliangiaceae
Granulo	:	Granulosicoccaceae
Erythro	:	Erythrobacteraceae
Sandara	:	Sandaracinaceae
Caldili	:	Caldilineaceae
Geobact	:	Geobacteraceae
Pisciri	:	Piscirickettsiaceae
Bacteri	:	Bacteriovoracaceae
Rhodocy	:	Rhodocyclaceae
Oceanos	:	Oceanospirillaceae
Rhodosp	:	Rhodospirillaceae
Halomon	:	Halomonadaceae
Hyphomi	:	Hyphomicrobiaceae
Rhizobi	:	Rhizobiaceae

Genus

Microb	:	Microbacterium
Arenic	:	Arenicella
Altero	:	Alteromonas
Mariba	:	Maribacter
Sphing	:	Sphingorhabdus
Polari	:	Polaribacter
Flavir	:	Flaviramulus
Litori	:	Litoribaculum
Fabiba	:	Fabibacter
Winogr	:	Winogradskyella
Kordia	:	Kordia
Crocin	:	Crocinitomix
Lewine	:	Lewinella
Sulfit	:	Sulfitobacter
Chondr	:	Chondromyces
Ulviba	:	Ulvibacter
Plesio	:	Plesiocystis
Gillis	:	Gillisia
Ekhidn	:	Ekhidna
Colwel	:	Colwellia
Achrom	:	Achromobacter
Aquaba	:	Aquabacterium
Marino	:	Marinobacter
Hyphom	:	Hyphomonas
Marini	:	Marinicella
Salini	:	Salinisphaera
Ilumat	:	Ilumatobacter
Paragl	:	Paraglaciecola
Loktan	:	Loktanella
Mesorh	:	Mesorhizobium
Halian	:	Haliangium
Granul	:	Granulosicoccus
Algiba	:	Algibacter
Tenaci	:	Tenacibaculum
Dokdon	:	Dokdonia
Portib	:	Portibacter
Owenwe	:	Owenweeksia
Sandar	:	Sandaracinus
Litori	:	Litorilinea
Geobac	:	Geobacter
Rueger	:	Ruegeria
Novosp	:	Novosphingobium
Nannoc	:	Nannocystis
Marixa	:	Marixanthomonas
Cycloc	:	Cycloclasticus
Octade	:	Octadecabacter
Amylib	:	Amylibacter
Haloba	:	Halobacteriovorax
Minicy	:	Minicystis
Azoarc	:	Azoarcus
Mariti	:	Maritimimonas
Olleya	:	Olleya
Crocei	:	Croceibacter
Oleisp	:	Oleispira
Halomo	:	Halomonas
Marita	:	Maritalea
Geojed	:	Geojedonia
Rhizob	:	Rhizobium
Reiche	:	Reichenbachiella

Species

Micr gins	:	*Microbacterium ginsengiterrae*
Alte geno	:	*Alteromonas genovensis*
Mari ulvi	:	*Maribacter ulvicola*
Sphi lito	:	*Sphingorhabdus litoris*
Pola reic	:	*Polaribacter reichenbachii*
Flav icht	:	*Flaviramulus ichthyoenteri*
Lito gwan	:	*Litoribaculum gwangyangense*
Wino unda	:	*Winogradskyella undariae*
Kord anta	:	*Kordia antarctica*
Croc cata	:	*Crocinitomix catalasitica*
Lewi agar	:	*Lewinella agarilytica*
Ples paci	:	*Plesiocystis pacifica*
Ekhi lute	:	*Ekhidna lutea*
Colw meon	:	*Colwellia meonggei*
Achr inso	:	*Achromobacter insolitus*
Aqua parv	:	*Aquabacterium parvum*
Mari flav	:	*Marinobacter flavimaris*
Hyph john	:	*Hyphomonas johnsonii*
Lewi anta	:	*Lewinella antarctica*
Mari lito	:	*Marinicella litoralis*
Sali dokd	:	*Salinisphaera dokdonensis*
Ilum cocc	:	*Ilumatobacter coccineum*
Para ocea	:	*Paraglaciecola oceanifecundans*
Lokt kore	:	*Loktanella koreensis*
Meso hawa	:	*Mesorhizobium hawassense*
Mari sala	:	*Marinobacter salarius*
Gran anta	:	*Granulosicoccus antarcticus*
Algi aest	:	*Algibacter aestuarii*
Tena sole	:	*Tenacibaculum soleae*
Lewi coha	:	*Lewinella cohaerens*
Dokd geni	:	*Dokdonia genika*
Port lacu	:	*Portibacter lacus*
Owen hong	:	*Owenweeksia hongkongensis*
Sand amyl	:	*Sandaracinus amylolyticus*
Dokd diap	:	*Dokdonia diaphoros*
Lito aero	:	*Litorilinea aerophila*
Rueg faec	:	*Ruegeria faecimaris*
Novo sedi	:	*Novosphingobium sediminicola*
Nann exed	:	*Nannocystis exedens*
Mari ophi	:	*Marixanthomonas ophiurae*
Cycl puge	:	*Cycloclasticus pugetii*
Lokt vest	:	*Loktanella vestfoldensis*
Octa anta	:	*Octadecabacter antarcticus*
Colw mari	:	*Colwellia maris*
Amyl mari	:	*Amylibacter marinus*
Halo mari	:	*Halobacteriovorax marinus*
Mini rose	:	*Minicystis rosea*
Azoa comm	:	*Azoarcus communis*
Colw chuk	:	*Colwellia chukchiensis*
Mari rapa	:	*Maritimimonas rapanae*
Aren chit	:	*Arenicella chitinivorans*
Wino wand	:	*Winogradskyella wandonensis*
Pola porp	:	*Polaribacter porphyrae*
Croc atla	:	*Croceibacter atlanticus*
Mari arct	:	*Maribacter arcticus*
Halo alka	:	*Halomonas alkaliphila*
Mari porp	:	*Maritalea porphyrae*
Geoj lito	:	*Geojedonia litorea*
Rhiz grah	:	*Rhizobium grahamii*

Supplementary Figure 10A

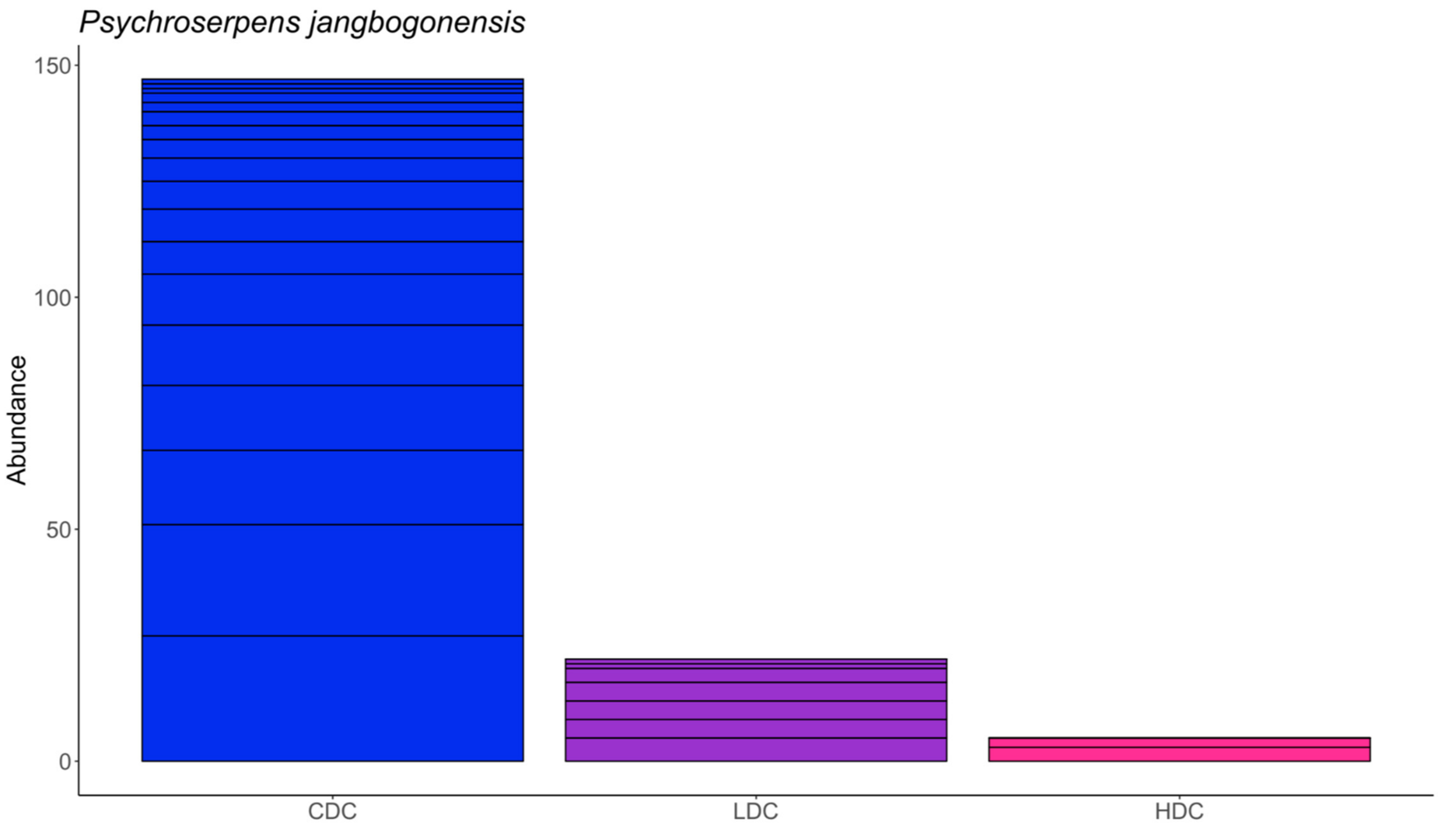

Supplementary Figure 10B

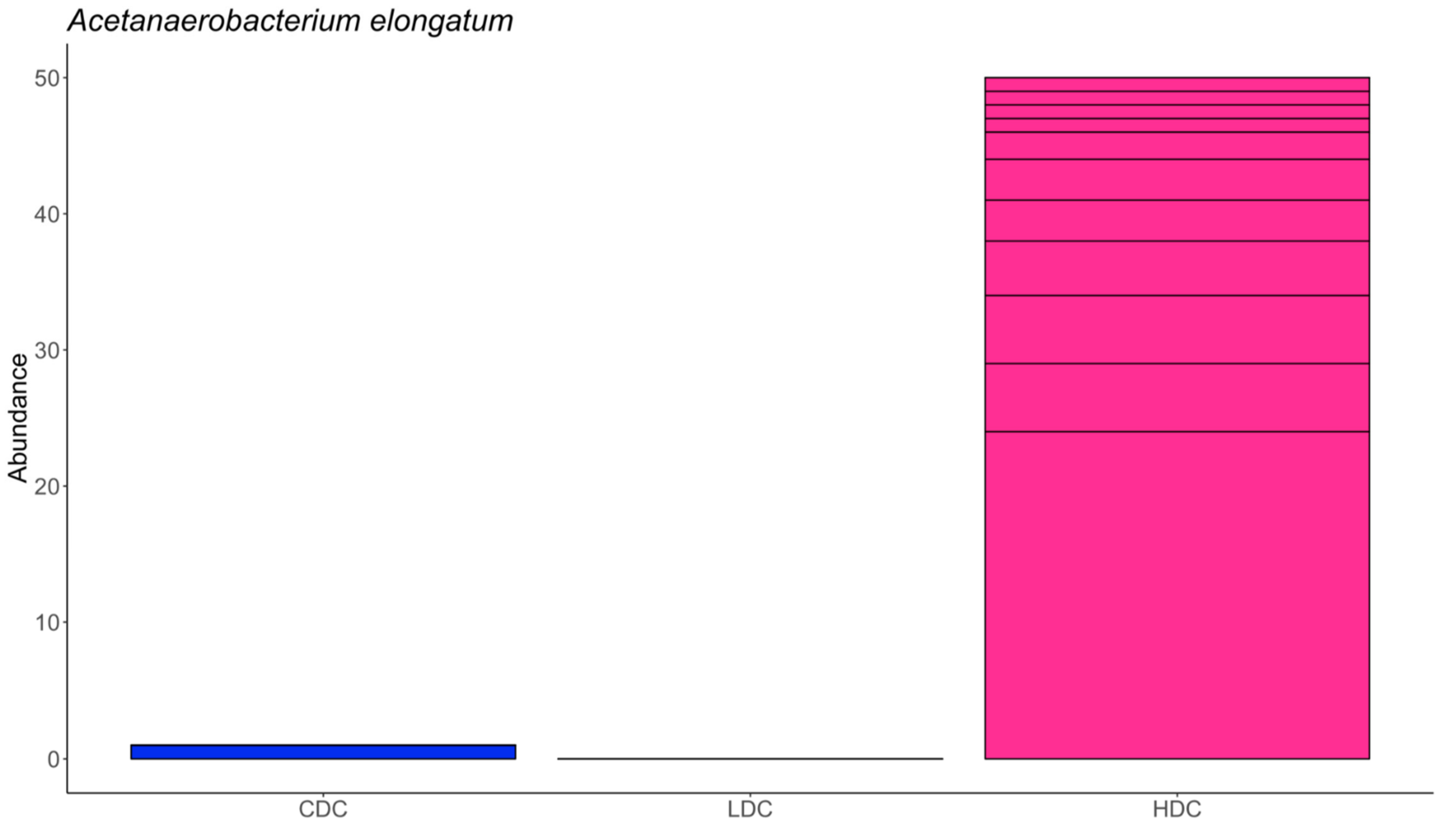

Supplementary Figure 10C

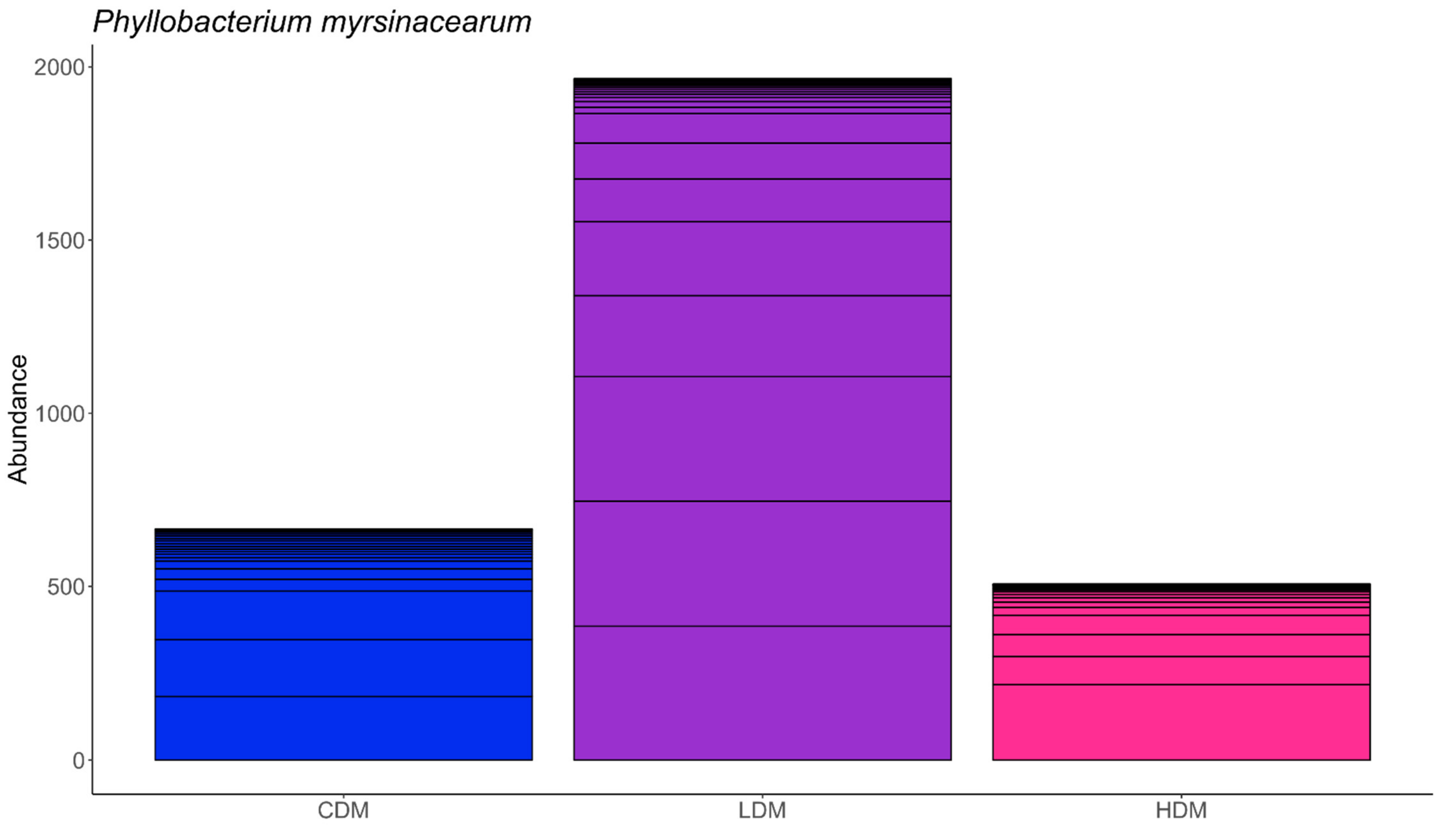

Supplementary Figure 10D

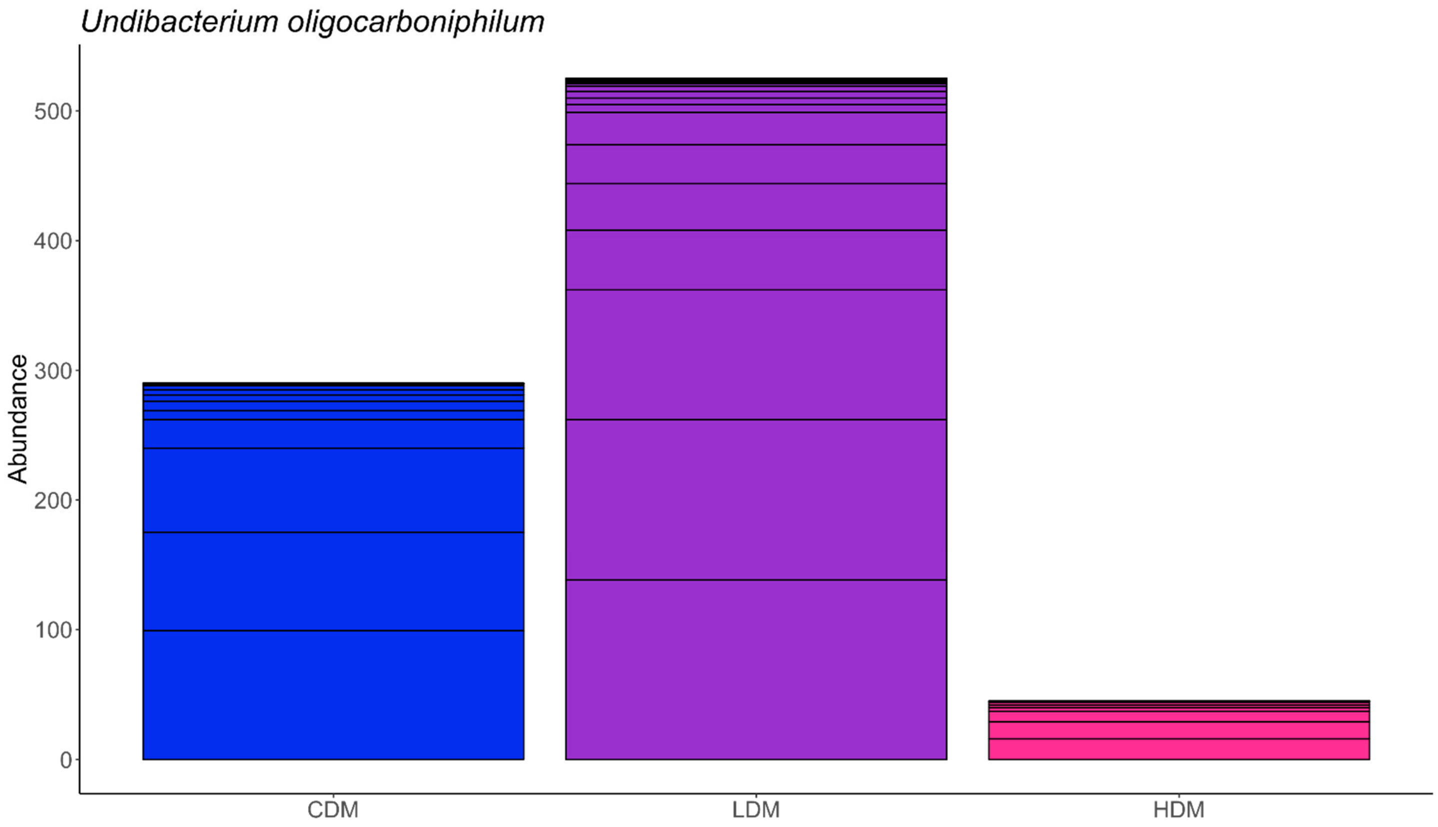

Supplementary Figure 10E

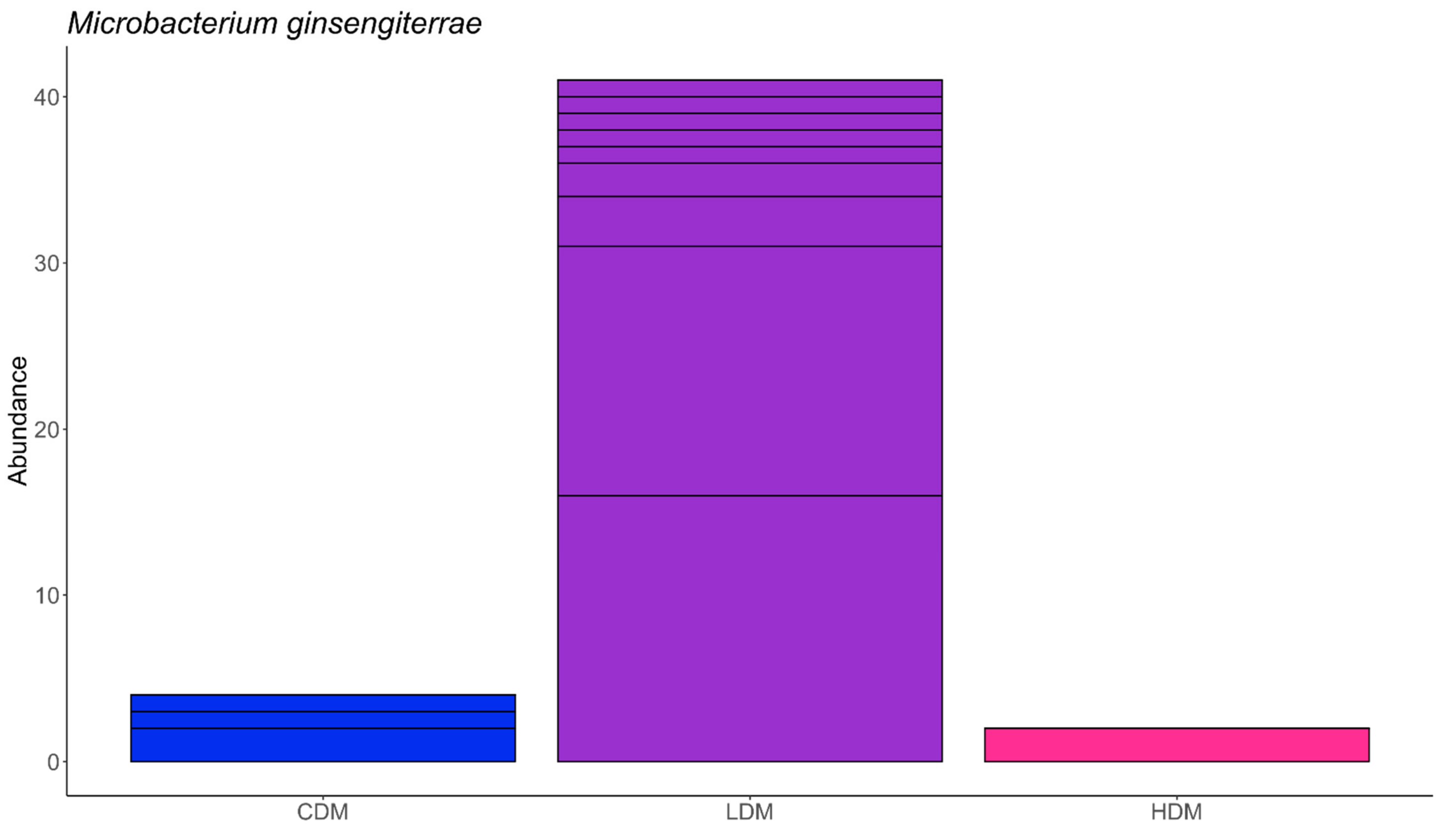

Supplement: FIGURE S1 — Boxplots showing the Faith’s phylogenetic diversity of the bacterial communities of distal intestinal content (A) and mucus (B). Different letters indicate statistically significant differences (P < 0.05) between the study groups. [file Data_Sheet_1.PDF]
